# Stretchable, Self‐Healing, and Bioactive Hydrogel with High‐Functionality N,N′‐bis(acryloyl)cystamine Dynamically Bonded Ag@polydopamine Crosslinkers for Wearable Sensors

**DOI:** 10.1002/advs.202404451

**Published:** 2024-07-19

**Authors:** Wei Shi, Hui Li, Jing Chen, Yern Chee Ching, Cheng Hock Chuah, Chengsheng Xu, Moran Liu, Jinyong Zhang, Kuan Yong Ching, Yongsheng Liang, Guanglin Li, Wei Tang

**Affiliations:** ^1^ Department of Chemical Engineering University of Malaya Lembah Pantai Kuala Lumpur 50603 Malaysia; ^2^ Key Laboratory of Human‐Machine‐Intelligence Synergic System Research Center for Neural Engineering Shenzhen Institute of Advanced Technology Chinese Academy of Sciences 1068 Xueyuan Road Shenzhen Guangdong 518055 China; ^3^ College of Big Data and Internet Shenzhen Technology University 3002 Lantian Road Shenzhen Guangdong 518118 China; ^4^ Department of Chemistry University of Malaya Lembah Pantai Kuala Lumpur 50603 Malaysia; ^5^ Foundation, Study and Language Institute University of Reading‐Malaysia Campus Persiaran Graduan, Kota Ilmu EduCity Iskandar Puteri Johor 79200 Malaysia

**Keywords:** health monitoring, hydrogel, self‐healing, sensor

## Abstract

Hydrogels present attractive opportunities as flexible sensors due to their soft nature and tunable physicochemical properties. Despite significant advances, practical application of hydrogel‐based sensor is limited by the lack of general routes to fabricate materials with combination of mechanical, conductive, and biological properties. Here, a multi‐functional hydrogel sensor is reported by in situ polymerizing of acrylamide (AM) with N,N′‐bis(acryloyl)cystamine (BA) dynamic crosslinked silver‐modified polydopamine (PDA) nanoparticles, namely PAM/BA‐Ag@PDA. Compared with traditional polyacrylamide (PAM) hydrogel, the BA‐Ag@PDA nanoparticles provide both high‐functionality crosslinks and multiple interactions within PAM networks, thereby endowing the optimized PAM/BA‐Ag@PDA hydrogel with significantly enhanced tensile/compressive strength (349.80 kPa at 383.57% tensile strain, 263.08 kPa at 90% compressive strain), lower hysteresis (5.2%), improved conductivity (2.51 S m^−1^) and excellent near‐infrared (NIR) light‐triggered self‐healing ability. As a strain sensor, the PAM/BA‐Ag@PDA hydrogel shows a good sensitivity (gauge factor of 1.86), rapid response time (138 ms), and high stability. Owing to abundant reactive groups in PDA, the PAM/BA‐Ag@PDA hydrogel exhibits inherent tissue adhesiveness and antioxidant, along with a synergistic antibacterial effect by PDA and Ag. Toward practical applications, the PAM/BA‐Ag@PDA hydrogel can conformally adhere to skin and monitor subtle activities and large‐scale movements with excellent reliability, demonstrating its promising applications as wearable sensors for healthcare.

## Graphical abstract

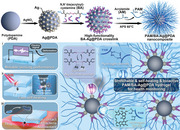

## 1 Introduction

Flexible sensors that can adhere to arbitrary surfaces and transduce mechanical deformations into electrical output signals have attracted tremendous attention and have been widely used for the applications of health monitoring, soft robots and human–machine interaction system.^[^ ^1^, ^2^, ^3^ ^]^ Compared with traditional flexible sensors composed of elastomeric matrix (e.g., polydimethylsiloxane (PDMS), polyurethane (PU), and Ecoflex) and conductive components (e.g., liquid metals, metal nanoparticles/nanowires, MXene),^[^ ^4^, ^5^ ^]^ hydrogels have emerged as a promising alternative for wearable electronics owing to their similarities to natural tissues and versatility in electrical, mechanical, and biofunctional engineering.^[^ ^6^, ^7^ ^]^ By rationally designing the hydrogel networks, hydrogel can be endowed with multiple combined conductive, mechanical, and biological properties.^[^ ^8^, ^9^ ^]^ Despite significant advances and prospects, hydrogel‐based electronics for personalized health monitoring that can synchronously meet the demands of good mechanical strength, high stretchability, desirable sensitivity, excellent reliability, and biocompatibility still remain numerous challenges.^[^ ^10^, ^11^ ^]^ For example, using conductive polymers, such as polypyrrole (PPy), polyaniline (PANI), poly(3,4‐ethylenedioxythiophene) (PEDOT), as the frameworks of hydrogel sensors can obtain high conductivity,^[^ ^12^ ^]^ but their biocompatibility, stretchability (intrinsic stiff and hydrophobic characteristics of the conductive polymers) and biodegradability need to be improved.^[^ ^13^ ^]^ Doping conductive agents into hydrogels as fillers would suffer phase separated instability, limited stretchability, or leakage issues.^[^ ^14^ ^]^ Moreover, most existing hydrogel sensors have complex components or complicated manufacturing process, restricting their wide applications.^[^ ^15^, ^16^ ^]^ Thus, developing advanced hydrogel sensor with combinations of stretchability, strength, sensitivity, biocompatibility, and ease of scalable fabrication is important for the next generation of wearable electronics.^[^ ^17^ ^]^

Meanwhile, to achieve long‐term conformal attachment on skin with longevity, hydrogel sensors with multi‐functional properties, including self‐adhesion, self‐healing as well as anti‐inflammation/anti‐bacterial capability have received considerable attention for biomedical applications.^[^ ^18^, ^19^, ^20^ ^]^ A variety of self‐adhesive and self‐healable hydrogel sensors were explored through synergistic reversible interactions of dynamic covalent (imine, acylhydrazone, disulfide bonds and borate ester bonds, etc.), non‐covalent (hydrogen bonding, ionic interaction, metal coordination, and host‐guest interaction, etc.) and irreversible covalent bonding (amide bond and carbon‐sulfide bond, etc.).^[^ ^21^, ^22^, ^23^ ^]^ Recently, Yu et al. developed a self‐adhesive, self‐healing, biocompatible strain/pressure hydrogel sensor by in situ polymerization of acrylamide (PAM) in the presence of polydopamine‐modified carbon nanotubes (PDA@CNTs). The abundant hydrogen bonding and π‐π stacking derived from the PDA ensure the PDA@CNT/PAM hydrogel sensor with an autonomous self‐healing behavior (97.3% healing efficiency after 6 h), and the CNTs enhance the electronic transmission capacity and mechanical properties (17.5 kPa tensile strength at >700% strain) of the hydrogel sensor.^[^ ^24^ ^]^ Furthermore, Zhang et al. developed a super stretchable, self‐healing, adhesive ionic conductive stress sensors based on cellulose nanofibrils (CNFs) modified phenylboronic acid‐ionic liquid (PBA‐IL)/acrylamide hydrogel. Owing to the multiple combined H‐bonds, electrostatic interactions, and borate ester bonds, the hydrogel sensor exhibited a tensile stress of 369.5 kPa ± 9.1 kPa with a self‐healing efficiency about 92% after 150 min.^[^ ^25^ ^]^ To further improve the healing speed and controllability, stimulus (e.g., heat, pH, near‐infrared (NIR) light) triggered self‐healing hydrogel sensors were investigated.^[^ ^26^ ^]^ Although tremendous progress, several issues remain unsolved. For instance, the mechanical strength of autonomous self‐healing hydrogel sensors is relatively weak.^[^ ^27^, ^28^ ^]^ Multiple synergistic interactions can improve the mechanical strength and self‐healing capability of hydrogel sensors, but their biocompatibility or stretchability might be compromised.^[^ ^29^ ^]^ Achieving optimal properties for a desired hydrogel sensors with excellent mechanical properties, high healing efficiency, and desirable bioactivity are generally difficult (Table S1, Supporting Information).^[^ ^29^, ^30^, ^31^ ^]^

**Scheme 1:**
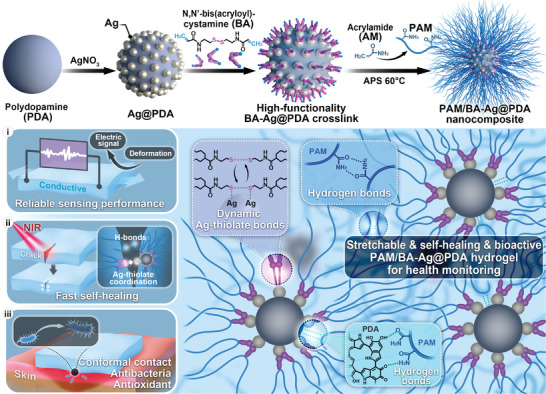
Schematic illustration of the PAM/BA‐Ag@PDA nanocomposite hydrogel for wearable sensors.

To address these, we propose a multi‐functional nanocomposite hydrogel (named PAM/BA‐Ag@PDA) with appealing characteristics as a strain sensor for human health monitoring by free radical polymerization of acrylamide (AM) with N,N′‐bis(acryloyl)cystamine (BA) modified silver‐doped polydopamine nanoparticles (BA‐Ag@PDA) (**Scheme** **1**). By introducing the crosslinkable BA‐Ag@PDA nanoparticles in the PAM hydrogel, improved mechanical strength with low hysteresis and good fatigue resistance can be achieved through the high‐functionality nanoparticle crosslinks and multiple interactions within the hydrogel networks. Moreover, the PAM/BA‐Ag@PDA hydrogel can be endowed with electrical sensitivity and NIR stimuli‐responsive ability due to the intrinsic conductive and photothermal ability of both Ag and PDA. Further, owing to the reversible crosslinks of BA‐Ag@PDA within the hydrogel network, by simultaneously involving abundant H‐bonds and Ag‐thiolate coordination, the PAM/BA‐Ag@PDA hydrogel can restore its original function after mechanical failure. Benefiting from the presence of catechol groups, the PAM/BA‐Ag@PDA hydrogel exhibits repeatable adhesiveness for conformal skin contact. Besides, due to the synergistic antibacterial effect of Ag with PDA and an antioxidant effect from PDA, the PAM/BA‐Ag@PDA is able to meet the increasing biologic requirements in future health monitoring.

## 2 Results and Discussion

### 2.1 Characterization of the PAM/BA‐Ag@PDA Hydrogel

**Figure 1:**
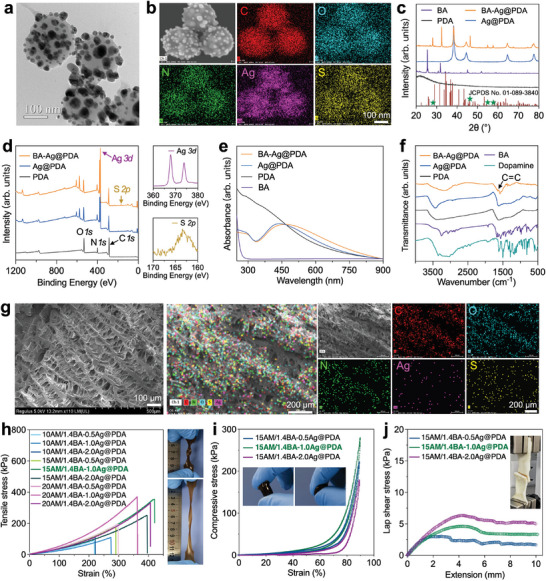
Characterization of the BA‐Ag@PDA nanoparticles. a) TEM image of the BA‐Ag@PDA nanoparticles. b) SEM and EDS mapping images of the BA‐Ag@PDA nanoparticles. c) XRD patterns of the BA, PDA, Ag@PDA, and BA‐Ag@PDA. d) XPS analysis of the PDA, Ag@PDA, and BA‐Ag@PDA nanoparticles. e) UV–vis–NIR absorption spectra of the BA, PDA, Ag@PDA, and BA‐Ag@PDA. f) FTIR spectra of the BA, dopamine, PDA, Ag@PDA, and BA‐Ag@PDA. g) SEM and EDS mapping images of the PAM/BA‐Ag@PDA network in the dry state. h) Tensile stress–strain curves of the PAM/BA‐Ag@PDA hydrogel with different contents of AM and Ag@PDA. Insert: Image of the 15AM/1.4BA‐1.0Ag@PDA group under twist and stretch condition. i) Compressive stress–strain curves and j) lap shear stress–strain curves of the PAM/BA‐Ag@PDA hydrogel with different contents ofAg@PDA.

The Ag@PDA nanoparticles were synthesized through in situ redox reaction between AgNO_3_ and PDA nanoparticles due to the presence of dihydroxyphenyl groups on PDA.^[^ ^32^ ^]^ Before Ag‐modification, the PDA nanoparticles displayed a uniform spherical shape with an average hydrodynamic diameter of 196 nm (Figures S1a and S2, Supporting Information), and the C, N, and O elements were homogeneously distributed in the nanoparticles. By contrast, the surface of Ag@PDA nanoparticles was deposited with large amounts of Ag dots. The elemental distribution map of Ag indicated the homogeneous presence of Ag on PDA (Figure S1b, Supporting Information). To further obtain highly functionalized networks, the Ag@PDA nanoparticles were modified by BA through the dynamic silver‐thiolate coordination interactions (named BA‐Ag@PDA), and then introduced as the crosslinkers within the PAM network. TEM image showed that the BA‐Ag@PDA nanoparticles had a lichee‐like morphology with an average diameter of 238 nm (**Figure** **1**a; Figure S2, Supporting Information). The corresponding SEM‐EDS mapping images of BA‐Ag@PDA indicated the C, N, O, Ag, and S elements were homogeneously distributed in the BA‐Ag@PDA (Figure 1b).

X‐ray diffraction (XRD) results indicated that the PDA had amorphous features with a broad peak around 2𝜃 = 24.2°.^[^ ^33^ ^]^ By contrast, obvious (111), (200), (220), and (311) diffraction peaks of metallic Ag at 38.2°, 44.1°, 64.3°, and 77.3° were observed in the XRD pattern of Ag@PDA and BA‐Ag@PDA.^[^ ^34^ ^]^ (Figure 1c). Noteworthy, the diffraction peaks of crystalline Ag phase in BA‐Ag@PDA were weakened than that of Ag@PDA after the incorporation of BA, whereas four new diffraction peaks (2θ) at 27.9°, 46.3°, 54.88°, and 57.54°, which could be indexed to the (021), (−123), (212), and (140) planes of Ag_2_S (JCPDS Card no. 01‐089‐3840) were observed in the BA‐Ag@PDA. Moreover, a specific diffraction peak located at 31.76° assigned to the diffraction peak of BA was found, in which the slight shift from 31.76° to higher angle of 32.32° might be ascribed to Ag‐thiolate interaction and the formation of Ag_2_S.

Consistent with these findings, the Raman spectra at 1345 and 1582 cm^−1^ in PDA, Ag@PDA and BA‐Ag@PDA corresponded to the stretching and deformation of aromatic rings in PDA backbone (Figure S3, Supporting Information).^[^ ^35^ ^]^ Moreover, X‐ray photoelectron spectroscopy (XPS) results of Ag@PDA and BA‐Ag@PDA at 367.8 eV (Ag 3d_5/2_) and 373.8 eV (Ag 3d_3/2_).^[^ ^36^, ^37^ ^]^ clearly indicated the existence of Ag on PDA (Figure 1d; Figure S4, Supporting Information). Meanwhile, a typical S 2p peak was detected in the XPS spectrum of BA‐Ag@PDA.^[^ ^38^, ^39^ ^]^ (Figure 1d). Further, in the UV–vis spectra, BA‐Ag@PDA showed a redshifted absorption peak from 440 to 462 nm arising from the surface plasmon resonance effect of Ag nanoparticles (Figure 1e; Figure S5, Supporting Information).^[^ ^40^, ^41^ ^]^ Next, we reconfirmed the existence of alkene on the Ag@PDA nanoparticles by Fourier transform infrared spectroscopy (FTIR). Compared with Ag@PDA, a new absorption peak at 1634 cm^−1^ ascribed to the alkene vibration was found in the BA‐Ag@PDA, further indicating the successful preparation of BA‐Ag@PDA (Figure 1f).

To demonstrate the advantages of BA‐Ag@PDA in constructing high‐performance PAM‐based hydrogel, we first investigated the gelation behavior, mechanical property, and self‐healing ability of the PAM hydrogel using BA as the crosslinker. We found that the precursor solution of 15% AM containing 1.4 mg mL^−1^ of BA could not be crosslinked within 12 min via thermally initiated polymerization (Figure S6, Supporting Information). Even though the gelation time was prolonged to 3 h, it was still unable to form fully crosslinked hydrogels. After demolding, a soft and partially crosslinked gel was observed for the PAM hydrogel. Moreover, the PAM hydrogel was fragile (9.66 kPa, Figure S7, Supporting Information), and no self‐healing phenomenon occurred in the PAM hydrogel under NIR irradiation (Figure S8, Supporting Information). SEM images indicated that the PAM hydrogel using BA as the crosslinker instead of the BA‐Ag@PDA nanoparticles displayed a collapsed and irregular network (Figure S9, Supporting Information). While the networks of PAM/BA‐Ag@PDA exhibited branched architectures, in which the elements of Ag and S belonged to BA‐Ag@PDA were mainly distributed in the linear regions (Figure 1g). Moreover, after swelling, a more compact and denser microporous network with pore size of about 10–50 µm was observed for the PAM/BA‐Ag@PDA than that of PAM. Since nano‐/microparticles can be introduced into polymer networks as high‐functionality crosslinks, providing tunable mechanical properties for hydrogels,^[^ ^42^, ^43^ ^]^ we systematically analyzed and optimized the properties of PAM/BA‐Ag@PDA hydrogel for their applications as wearable sensor. Detailed preparation information of the PAM/BA‐Ag@PDA hydrogels with different contents of AM, BA, and Ag@PDA is summarized in Table S2 (Supporting Information).

Tensile stress–strain curves of the PAM/BA‐Ag@PDA hydrogels showed that a higher amount of AM resulted in a greater stretchability of hydrogel. Increasing the amount of BA‐Ag@PDA, the tensile stress of hydrogel first increased and then decreased. According to the definition of hydrogel networks, the PAM/BA‐Ag@PDA hydrogel networks belong to the category of unconventional polymer networks, in which both the high‐functionality architectures (multiple polymer chains can be interconnected at the crosslink of BA‐Ag@PDA nanoparticles) and multiple interactions (physical and reversible interactions) among polymer chains exist in the network.^[^ ^42^, ^43^, ^44^ ^]^ (Scheme 1). In the dry state of a conventional polymer network, the end‐to‐end distances of a polymer chain at the relaxed and fully stretched states are defined as $\sqrt{N}$ b and Nb, respectively, where N is the number of Kuhn monomers in each polymer chain, b is the length of each Kuhn monomer. Therefore, the stretch limit of the polymer chains (λ_lim_) in a dry polymer network can be calculated as λ_lim_ = $\mathrm{Nb}÷\sqrt{N}$ b = $\sqrt{N}$. Although the PAM/BA‐Ag@PDA hydrogel networks are a kind of multimodal polymer networks, the longest polymer chains in PAM/BA‐Ag@PDA hydrogel can maintain its integrity up to the stretch limit, which can be calculated as: λ_lim_ = $\sqrt{N_{max}}\;÷\;$λ_s_, where N_max_ is the number of Kuhn monomers on the longest polymer chain, and λs is the effect of swelling on the λ_lim_. Following this principle, the stretchability of PAM/BA‐Ag@PDA hydrogel is closely related with the number of Kuhn monomers on the longest polymer chain. Correspondingly, with a constant amount of BA‐Ag@PDA, the strain of PAM/BA‐Ag@PDA hydrogel increases with the increase of AM monomer amount. Moreover, at a constant amount of AM monomer, a higher amount of BA‐Ag@PDA provides higher density of functionality crosslinks per unit volume in the hydrogel network, causing decreased number of Kuhn monomers in each polymer chain. Therefore, the tensile strain of PAM/BA‐Ag@PDA hydrogel at a high amount of BA‐Ag@PDA was significantly decreased (Figures S10–S14, Supporting Information). Our experimental observations are in good agreement with the theoretical analyses.

Tensile strength is commonly defined as the stresses at which the ultimate tensile failure occurs in the uniaxial tensile test, which can be evaluated as σ_f_ = m_f_f_f_, where f_f_ is the force required to fracture a single polymer chain, m_f_ are the numbers of simultaneously fractured polymer chains per unit area of the polymer network at the deformed states. The design principle of strong hydrogels is to form substantial number of polymer chains in per unit area of polymer network to fracture simultaneously.^[^ ^42^ ^]^ A greater number of polymer chains fracture simultaneously gives a higher tensile strength of the hydrogels under larger deformation. In the network of PAM/BA‐Ag@PDA hydrogel, there were multiple polymer chains bridging between two adjacent BA‐Ag@PDA crosslinks. At different ratios of AM, BA‐Ag@PDA, the number and lengths of polymer chains between adjacent crosslinks as well as the density of dynamic Ag‐thiolate coordination between polymer chains and crosslinks were different, thereby endowing the PAM/BA‐Ag@PDA hydrogel with different mechanical strength. Based on the strength principle and our experimental data, we found that without enough BA‐Ag@PDA crosslinks in the hydrogel networks, the AM monomers were mainly polymerized into long PAM polymer chains, resulting in limited number of polymer chains but prolonged polymer chains per unit volume. Therefore, a compromised tensile strength but excellent tensile strain was observed for the PAM/BA‐Ag@PDA hydrogels containing a low concentration of BA‐Ag@PDA (Figures S10 and S11, Supporting Information). While, increasing the concentration of BA‐Ag@PDA, the tensile strength of PAM/BA‐Ag@PDA hydrogels first increased and then decreased. Notably, at the highest concentration of BA‐Ag@PDA, the tensile strength of PAM/BA‐Ag@PDA hydrogels was significantly decreased at either low or high concentration of AM monomers (Figures S10 and S14, Supporting Information), indicating that a high crosslinks density might result in nonuniform lengths of polymer chains in the PAM/BA‐Ag@PDA networks. As the hydrogels undergo deforms, the short polymer chains and long polymer chains are successively fractured but not simultaneously fractured.

Among them, the PAM/BA‐Ag@PDA hydrogel containing 20% AM, 1.0 mg mL^−1^ of BA and 2.0 mg mL^−1^ of Ag@PDA (named 20AM/1.0BA‐2.0Ag@PDA) achieved highest tensile strength (up to 789.89 kPa) with a maximal stretching ratio of 1154.37% (Figures S10 and S12, Supporting Information). Despite attractive tensile properties, the 20AM/1.0BA‐2.0Ag@PDA showed unsatisfactory reversibility with obvious permanent deformation after the stress was removed (Figure S15, Supporting Information). A hysteresis (19.3%) was observed in the loading‐unloading curve (Figure S16, Supporting Information). The significant hysteresis would cause unreliable output electrical signal, impeding the practical use of hydrogel sensor. Comparatively, the PAM/BA‐Ag@PDA hydrogels containing 15% AM, 1.4 mg mL^−1^ of BA and 1.0 mg mL^−1^ of Ag@PDA (named 15AM/1.4BA‐1.0Ag@PDA) displayed good tensile stress/stretchability (349.80 kPa at 383.57% strain, Figure 1h and Figure S13, Supporting Information), low hysteresis (5.2%, Figure S16, Supporting Information) and desirable stability (Figure S17, Supporting Information), guaranteeing its potential as strain sensor. Further, the 15PAM/1.4BA‐1.0Ag@PDA hydrogel remained undamaged when compressed to a large strain of 90% with a maximal compressive stress of 263.08 kPa (Figure 1i). When applying 30%, 50%, and 70% compression strains, the 15AM/1.4BA‐1.0Ag@PDA hydrogel also showed neglectable recovery loss, revealing its excellent compression resilience under various compression strains (Figure S18, Supporting Information). In addition, the 15AM/1.4BA‐1.0Ag@PDA hydrogel could directly adhere to organs or tissues without assistance of tapes (Figure S19, Supporting Information). As determined by lap shear test using pork skin as adherends, the 15AM/1.4BA‐1.0Ag@PDA hydrogel showed an adhesion strength of 5.00 kPa (Figure 1j; Figure S20, Supporting Information). Collectively, the PAM/BA‐Ag@PDA hydrogel containing 15% AM, 1.4 mg mL^−1^ of BA and 1.0 mg mL^−1^ of Ag@PDA with optimal mechanical properties and good resilience was adopted for the application of strain sensor.

### 2.2 Sensing Properties of the PAM/BA‐Ag@PDA Hydrogel Based Resistive Strain Sensor

**Figure 2:**
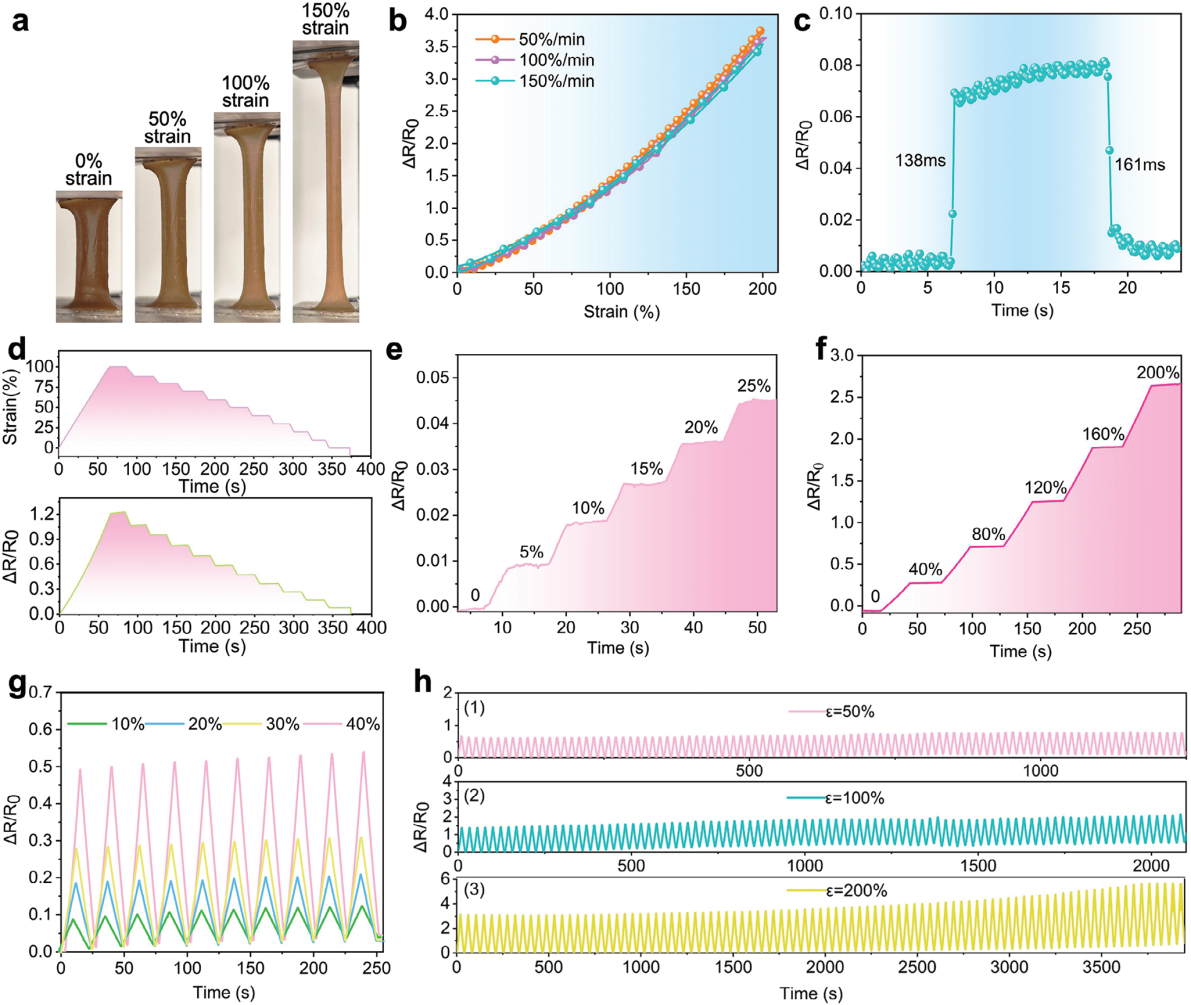
Strain sensing properties of the PAM/BA‐Ag@PDA hydrogel‐based resistive strain sensors. a) Photographs of the PAM/BA‐Ag@PDA hydrogel sensor to strains during the uniaxial stretching test. b) Relative resistance changes (ΔR/R_0_ signal) of the PAM/BA‐Ag@PDA hydrogel sensor at different stretching speeds (50% min^−1^, 100% min^−1^, 150% min^−1^) for one cycle. c) Response and recovery time of the PAM/BA‐Ag@PDA hydrogel sensor under small tensile strain. d) Dynamic response of the PAM/BA‐Ag@PDA hydrogel sensor under a series of unloading step‐down strains from 100% to the initial state. e,f) Relative resistance changes of the PAM/BA‐Ag@PDA hydrogel sensor under increasing strain increments of 5% and 40%. g) Relative resistance changes of the PAM/BA‐Ag@PDA hydrogel sensor during cyclic tensile test at 10%, 20%, 30%, and 40% strain (strain frequency: 0.04 Hz). h) Relative resistance changes of the PAM/BA‐Ag@PDA hydrogel sensor at 50%, 100%, and 200% strain for 100 cycles (stretching–releasing speed: 120 mm min^−1^).

Next, we measured the conductivity of PAM/BA‐Ag@PDA hydrogel. Notably, the conductivity of PAM/BA‐Ag@PDA hydrogel was 2.51 S m^−1^, which was 4.5‐fold higher than that of PAM (Figure S21, Supporting Information). To quantitatively characterize the flexible strain sensor based on PAM/BA‐Ag@PDA hydrogel, the hydrogel sensor was directly connected to an impedance analyzer to record the real‐time change in resistance under various stimuli from a universal testing machine. As depicted in **Figure** **2**a, the dumbbell‐shaped hydrogel was stretched from 0% to 200% without breakage, displaying excellent stretchability. The relative change in resistance of the hydrogel‐based resistive strain sensor was generated at loading/unloading speeds of 50% strain per min to 150% strain per min with the same tensile strain (200%), as shown in Figure 2b. The output curves were stable and monotonic, showing high linearity and positive correlation with tensile strain. The sensitivity of the strain sensor was evaluated by gauge factor (GF), which is defined as $\mathrm{GF}=\frac{\Delta R/R_{0}}{\varepsilon }\times 100\%$, where ΔR and R_0_ represent the change in resistance and the initial resistance of the hydrogel strain sensor respectively.^[^ ^45^ ^]^ The GF value for the conductive hydrogel across a 200% strain range was approximately 1.86, indicating its good potential sensitivity as a strain sensor. The response time was another key factor for evaluating the performance of a sensor when detecting rapid vibrations. Figure 2c shows that the sensor provided a fast response to both stretching and relaxing processes. The response time and recovery time were 138 and 161 ms, respectively, enabling the device to sense real‐life human motions timely, such as human–machine interfacing,^[^ ^46^ ^]^ subtle throat movement,^[^ ^47^ ^]^ and muscle contracting,^[^ ^48^ ^]^ etc. The slight drift in peak plateau data is mainly associated with the viscoelastic effect of hydrogels.^[^ ^25^, ^46^ ^]^ When the velocity of loading is high, residual deformation occurs, leading to a gradual upward drift in resistance. This effect is particularly pronounced during response time testing, where the loading speed reaches as high as 1100 mm min^−1^.

Step and hold tests were conducted to evaluate the strain sensor stability and limit of detection (LOD) with different magnitudes of loading/unloading processes. As shown in Figure 2d, a 10% per step release from 100% strain to the initial state resulted in the sensor's resistance change behavior responding remarkably fast and robustly to the applied strain. Additionally, similar to the unloading process, the sensor also exhibited outstanding stability and excellent reliability whether under small (5%) or large (40%) stretching process step by step. Figure 2e,f shows the response signal trend of the PAM/BA‐Ag@PDA hydrogel sensor as a regular step‐like change with negligible overshoot. The dynamic responsive behaviors of the sensor under cyclic loading were displayed in Figure 2g, which exhibited a uniform and consistent output to the cyclic loading applied. The responses to different strains were almost identical except for the peak magnitude of the output curves with a noise‐free and stable strain response observed from 10% to 40%.

For practical applications, strain sensors also need to be highly stretchable to achieve optimal sensing performance, such as accommodating up to 100% strain in human joint motion.^[^ ^49^ ^]^ Furthermore, the ability of strain sensors to meet the demand of large deformations enhances their wearability, preventing discomfort from constriction, especially when applied to complex biological surfaces (>50% strain).^[^ ^50^ ^]^ Accordingly, the durability and fatigue resistance of the hydrogel‐based strain sensor were also experimentally tested by continuously stretching/releasing strains of 50%, 100%, and 200%. As shown in Figure 2h, ΔR/R_0_ increased as the strain increased, and the sensor also adapted well to continuous different degrees of strain. The real‐time resistance variation signals maintained nearly identical values with only a slight attenuation observed after 100 cycles for different degrees of strain, revealing its high durability in long‐term usage.

### 2.3 Sensing Properties of the Self‐Healed PAM/BA‐Ag@PDA Hydrogel

**Figure 3:**
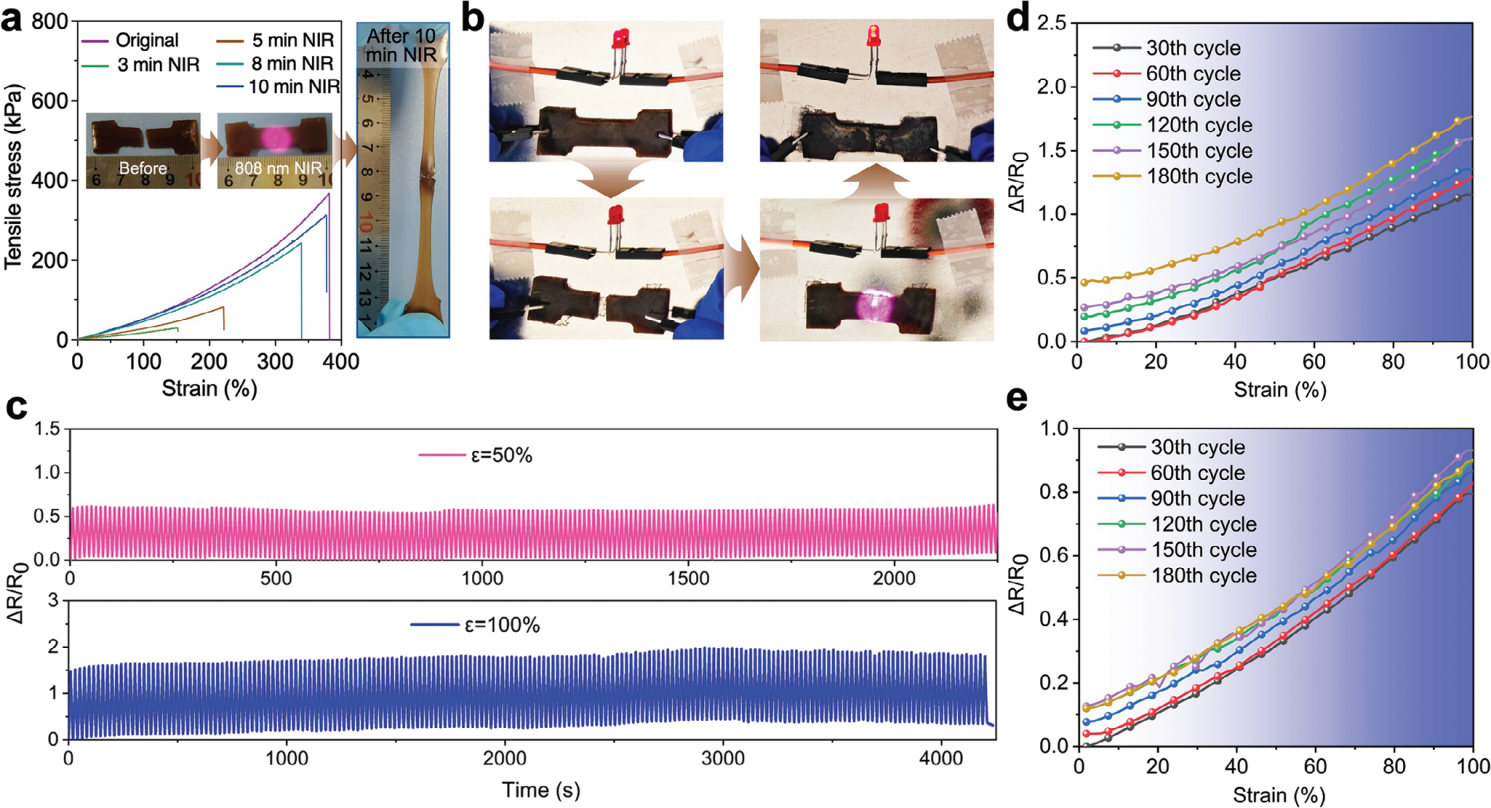
a) Self‐healing behavior of the PAM/BA‐Ag@PDA hydrogel after NIR irradiation. b) The LED electrical performance of the self‐healed PAM/BA‐Ag@PDA hydrogel. c) Relative resistance changes of the self‐healed PAM/BA‐Ag@PDA hydrogel at 50% and 100% strain for 200 consecutive cycles (stretching–releasing speed: 120 mm min^−1^). Relative resistance changes of the d) original and e) self‐healed PAM/BA‐Ag@PDA hydrogel at 30th, 60th, 90th, 120th, 150th, and 180th cycles in the strain range of 0–100%.

The self‐healing ability of material to recover from physical damage is another attractive feature for wearable sensor with improved durability and longevity. Owing to the reversible Ag‐thiolate coordination,^[^ ^51^, ^52^ ^]^ the PAM/BA‐Ag@PDA exhibited great NIR‐responsive self‐healing performance during the cutting‐healing process (**Figure** **3**a). We measured the tensile property of the self‐healed PAM/BA‐Ag@PDA hydrogel. After 10 min of NIR irradiation, the healed hydrogel could withstand 364.2% strain and a tensile stress of 282.53 kPa (Figure 3a; Figure S22a, Supporting Information). The tensile stress–strain data indicated that the healing efficiency of the PAM/BA‐Ag@PDA hydrogel was related to the NIR exposure time (Figure S22b, Supporting Information). Moreover, the Ag@PDA nanoparticles could also provide dynamic interactions with the BA‐PAM polymer chain via the thiolate‐metal coordination, endowing a distinctive self‐healing function between the PAM and PAM/BA‐Ag@PDA hydrogel (Figure S8, Supporting Information).

In the light‐emitting diode (LED) circuit experiment, the brightness of LED bulb immediately went off when the PAM/BA‐Ag@PDA sensor was cut off. After NIR irradiation for 8 min, the red LED was lighted up again due to the self‐healing ability of the PAM/BA‐Ag@PDA hydrogel (Figure 3b). To further confirm its reliability and durability, the sensing performances of the healed PAM/BA‐Ag@PDA hydrogel during cyclic tensile test were investigated at 50% and 100% strain. As shown in Figure 3c, the healed PAM/BA‐Ag@PDA hydrogel still maintained a good stability with negligible decay of resistance after 200 consecutive stretching–releasing cycles. Correspondingly, the output ΔR/R_0_ signals of the original and self‐healed PAM/BA‐Ag@PDA hydrogel at 100% strain of different cycles (30th, 60th, 90th, 120th, 150th, and 180th) were shown in Figure 3d,e, in which only a slight upward drift was observed between each cycle. The main reason for the drift in various cycles was that the plastic deformation of hydrogel existed under prolonged large deformation.^[^ ^53^, ^54^ ^]^ In terms of the cyclic tensile test at 200% strain, the relative resistance amplitude grew larger after 100 stretch cycles, which was mainly due to the cracks appeared in the healed hydrogel under large deformation (Figure S23, Supporting Information).

### 2.4 Antioxidant, Antibacteria, and Biocompatibility of the PAM/BA‐Ag@PDA Hydrogel

**Figure 4:**
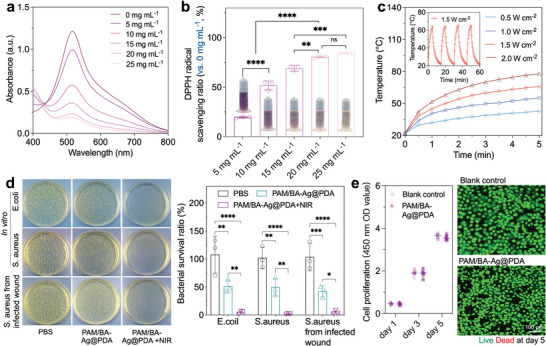
a) UV–vis absorption spectra of the DPPH free radicals incubated with different concentrations of PAM/BA‐Ag@PDA hydrogel. b) DPPH radical scavenging percentage of the PAM/BA‐Ag@PDA hydrogel at different concentrations. c) Photothermal efficiency of the PAM/BA‐Ag@PDA hydrogel at different NIR power densities. Insert image: Photostability of the PAM/BA‐Ag@PDA hydrogel after four on/off NIR irradiation (1.5 W cm^−2^) cycles. d) Antibacterial activity of the PAM/BA‐Ag@PDA hydrogels with or without NIR irradiation (808 nm, 1.5 W cm^−2^, 3 min). e) Biocompatibility of the PAM/BA‐Ag@PDA hydrogel determined by CCK‐8 and Live/Dead assay.

As a skin‐attachable electronic for health detection, there is a clinical need for improved the bioactivity and biocompatibility of sensors to prevent inflammation, microbial infections, and potential toxicity. Dopamine, the natural polyphenol existing in human, fruits, and vegetables, contains abundant phenolic hydroxyl groups that can react with free radicals to terminate the oxidation via hydrogen transfer and single electron transfer reactions, possessing outstanding antioxidant properties.^[^ ^55^, ^56^, ^57^ ^]^ To verify these functions, the antioxidant effect, antibacterial activity, and cell cytotoxicity of the PAM/BA‐Ag@PDA hydrogel were evaluated by 2,2‐diphenyl‐1‐picrylhydrazyl (DPPH) free radical scavenging assay, bacterial killing test, and Live/Dead staining, respectively. As indicated in **Figure** **4**a, the UV–vis absorbance of DPPH radical at 517 nm gradually deceased in response to increasing concentration of PAM/BA‐Ag@PDA hydrogel. At a concentration of 20 mg mL^−1^, 80.53% of the DPPH radical was eliminated by the PAM/BA‐Ag@PDA hydrogel (Figure 4b). Moreover, due to the conjugated π–π structure in PDA that electron leap can occur under 808 nm NIR irradiation, the PAM/BA‐Ag@PDA exhibited excellent photothermal conversion ability under NIR irradiation. Increasing the power density of NIR, an increased photothermal conversion performance was observed. With a NIR power density of 1.5 W cm^−2^, the temperature of PAM/BA‐Ag@PDA could reach ≈65.9 °C in 5 min, and the photothermal conversion capability maintained stable after 4 heating‐cooling cycles (Figure 4c).

The antibacterial abilities of PAM/BA‐Ag@PDA hydrogels were investigated by bacterial colony‐forming units (CFUs) counting test on agar plate (Figure 4d). In vitro antibacterial tests proved a significant antibacterial activity of PAM/BA‐Ag@PDA to both *Escherichia coli* (*E. coli*, Gram‐negative bacteria) and *Staphylococcus aureus* (*S. aureus*, Gram‐positive bacteria). Compared with PBS, 48.54% of *E. coli* and 50.03% of *S. aureus* were killed by PAM/BA‐Ag@PDA. Moreover, owing to the synergistic effects of bacteria capture, Ag ion release and photothermal conversion from Ag@PDA, the NIR‐treated PAM/BA‐Ag@PDA hydrogel exhibited a further enhanced antibacterial effect. The bacteria‐killing efficiency of NIR‐treated PAM/BA‐Ag@PDA for *E. coli* and *S. aureus* were calculated to be 95.74% and 98.27%, respectively. In accordance with the in vitro observation, the PAM/BA‐Ag@PDA also exhibited a potent antibacterial activity in a *S. aureus*‐infected rat full‐thickness skin defect model. The levels of viable bacteria for control, PAM/BA‐Ag@PDA and NIR‐treated PAM/BA‐Ag@PDA were 103.56%, 42.22%, and 4.21%, respectively, reconfirming the excellent and broad‐spectrum antibacterial activity of PAM/BA‐Ag@PDA. Additionally, CCK‐8 results indicated that the PAM/BA‐Ag@PDA hydrogel could support the normal proliferation of cells (Figure 4e). There was no significant difference in cellular proliferation between the PAM/BA‐Ag@PDA and control. Consistently, Live/Dead staining of the cells co‐incubated with PAM/BA‐Ag@PDA hydrogel showed a similar green fluorescence to the control group, reconfirming that the PAM/BA‐Ag@PDA sensor had no side effects on cytocompatibility.

### 2.5 Practical Applications for Human Movement Detection and Human–Machine Interface

**Figure 5:**
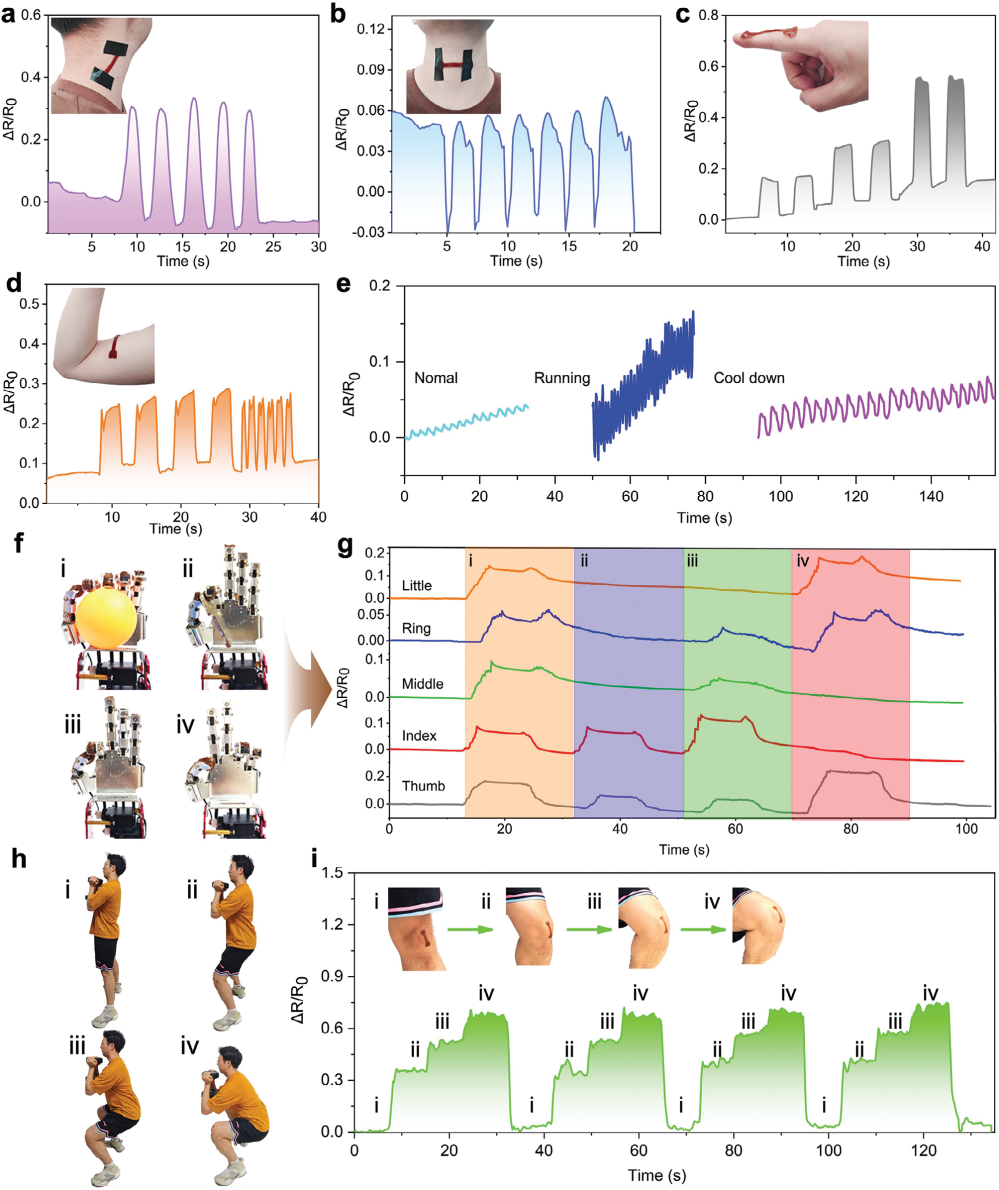
Detecting strain changes in human movement for healthcare monitor and human–machine interface. Relative resistance responses of the PAM/BA‐Ag@PDA based resistive strain sensor to: a) neck twisting, b) subtle throat movements, c) finger bending with different amplitudes, d) biceps contracting, e) real‐time sensing of breathing during standing (normal), running, and recovery by attaching the sensor to the abdomen. f) Photograph of the robot hand integrated with five hydrogel‐based resistive strain sensors on the back of fingers. g) Electrical signals of the robot hand for different gestures. h) Photograph of the application of the PAM/BA‐Ag@PDA hydrogel sensor to monitor knee‐joint activity during the squats. i) Real‐time relative resistance changes of the PAM/BA‐Ag@PDA hydrogel sensor used for large deformation of the knee‐joint.

In our daily life, various human activities necessitate strain sensors to recognize distinct degrees of skin strain, joint bending, etc. The excellent strain sensing properties of the PAM/BA‐Ag@PDA hydrogel‐based resistive strain sensors enable their use in various scenarios. As depicted in **Figure** **5**a, the hydrogel sensor was adhered to a volunteer's neck to monitor his neck shake to left and right shoulder. The highly reversible and repeatable response signals were recorded when the volunteer tilted his head, showcasing the potential application of this technique for aiding cervical syndrome patients in rehabilitation. Similarly, Figure 5b demonstrates the application of the PAM/BA‐Ag@PDA sensor on the laryngeal node to measure the throat's movement up and down during swallowing activities. The sensor maintained a stable response during the act of drinking water, demonstrating the potential application of this sensor in addressing oropharyngeal dysphagia resulting from stroke or other diseases. As seen in the inset of Figure 5c, a single PAM/BA‐Ag@PDA sensor was adhered to the index finger joint to sense the bending angle. When the index finger was continuously bent at 30°, 60°, and 90° and held for approximately 3 s, the recorded signal showed distinct ladder‐shaped resistance changes with the gradually increased bending angle. The results indicated that the PAM/BA‐Ag@PDA sensor was promising for use in motor function evaluation with good stability. In Figure 5d, the hydrogel sensor was employed to monitor biceps contraction and relaxation according to the variation of resistance with good repeatability. Additionally, changes in contracting frequency also resulted in changes in resistance with the same frequency, highlighting the sensor's ability to real‐time and accurately record muscle movements. The most notable characteristic of the obtained sensor was its high sensitivity to various vibration stimuli. By attaching the hydrogel sensor onto the chest of a volunteer, the periodic expansion and contraction states were captured precisely. Figure 5e shows that the sensor maintained a stable response during standing, running, and recovery states. From the normal state to running, both the velocity and intensity of breath waveforms sharply increased, indicating that volunteers required increasing amounts of oxygen during exercise to support the activity and metabolize the produced carbon dioxide. As the subject gradually cooled down, the breath waveform began to converge toward the normal state. It holds significant potential for use in athlete training, sports competitions, as well as monitoring respiratory‐related diseases.

As a further proof‐of‐concept demonstration of human–machine interaction, the hydrogel sensors were attached to the back surface of a robot fingers to measure the time‐dependent single signal of each finger, as shown in Figure 5f. When the robot fingers moved, data regarding the movements were captured, which could be aggregated into a sophisticated array of electrical signals representing various gestures. As displayed in Figure 5g, the finger data channels were independent, and each electrical signal included a set of crests and troughs, which could reflect the position of the fingers. The hydrogel sensors could accurately identify different motions when the robot hand grasped two different objects: the grip of table tennis required bending all five fingers, resulting in peak signals in all five channels of relative resistance change. Simultaneously, the grip of a key involved only the thumb and index finger, thus corresponding strain hydrogel sensors registered changes in their output signals. Sign language such as “ok” and “yeah” could be distinguished by the difference in the fast‐changing signal details; signal changes in all channels were clearly observed when the robot hand gestured. The exceptional ability to enable the robot hand sense various gestures endows this hydrogel strain sensor with promising potential for human–machine interface and sign language communication.^[^ ^58^ ^]^ Moreover, to ensure accurate measurement under large deformation, we tested the PAM/BA‐Ag@PDA hydrogel sensor's capability to monitor large‐scale bending‐related human knee‐joint movements. As shown in Figure 5h,i, when the volunteer changed his transitions from a standing position to a full squat, the knee joint's bending angle progressively increased from 0° to over 90°. Synchronously, the strain sensor converted the knee‐joint movements into resistance output signals with excellent repeatability and reliability.

## 3 Conclusion

In summary, PAM/BA‐Ag@PDA hydrogel was developed as biofunctional sensor for health monitoring. Various ratios of PAM, BA, Ag@PDA were measured, and the optimal combination was chosen as the strain sensor with excellent tensile properties and resilience. In vitro adhesion tests showed the hydrogel sensor had good adhesion to tissue. Abundant H‐bonds and Ag‐thiolate coordination within the hydrogel matrix also provided the self‐healing property. The PAM/BA‐Ag@PDA sensor was antioxidative, antibacterial, and biocompatible by evaluation of DPPH free radical scavenging assay, bacterial killing test, and Live/Dead staining. All of the above are important for the hydrogel sensor in wearable applications. Characterization of the hydrogel sensor was conducted, which revealed the sensor had desired sensitivity, high linearity, low hysteresis, and good repeatability. Corresponding applications of human motion detection and human‐machine interfaces were conducted to show the potential of PAM/BA‐Ag@PDA hydrogel for a variety of applications in wearable sensors and electronic skins.

## 4 Experimental Section

### Synthesis and Characterization of Polydopamine Nanoparticles (PDA NPs)

Two millimeters NH_4_OH (28–30%, Sigma, 221 228) was mixed with 40 mL ethanol and 90 mL deionized water under mild stirring for 1.5 h. Dopamine hydrochloride (0.5 g) (Sigma, H8502) was dissolved in 10 mL deionized water and then added into above mixture dropwisely. After reacting at 800 rpm for 24 h, PDA NPs were collected by centrifugation at 12 000 rpm for 15 min. The PDA NPs were then washed (thrice with ethanol and thrice with water) and stored at 4 °C until use.

### Synthesis and Characterization of Ag@PDA Nanoparticles (Ag@PDA NPs)

NH_4_OH was added into 5% (w/v%) AgNO_3_ （Aladdin, S116265）solution until the brown precipitates were completely eliminated, followed by reacting with 1% (w/v%, in deionized water) of PDA NPs at room temperature (RT) in the dark for 1 h. Then, the obtained Ag@PDA NPs were washed thrice with ethanol and deionized water alternately, and collected via centrifugation at 12 000 rpm for 15 min. The Ag@PDA NPs were kept at 4 °C until used. The morphologies of BA‐Ag@PDA, Ag@PDA, and PDA nanoparticles were investigated by field emission scanning electron microscopy (FESEM, SU8220, Hitachi) and transmission electron microscopy (TEM, JEM‐3200FS, JEOL). Elemental analysis of BA‐Ag@PDA, Ag@PDA, and PDA nanoparticles was confirmed by an Energy Dispersive Spectrometer (EDS, Oxford AZtec X‐Max 150). The crystal structure of BA‐Ag@PDA, Ag@PDA and PDA was investigated via Powder X‐ray diffraction (XRD, D8 discover, Bruker). Chemical structure of BA‐Ag@PDA, Ag@PDA, and PDA was characterized by X‐ray photoelectron spectroscopy (XPS, ESCALAB 250Xi, Thermo Scientific), Fourier transform infrared spectroscopy (FTIR, Nicolet iS50, Thermo Scientific), UV–vis spectrophotometer (UV‐2600, Shimadzu) and The Raman spectra (LabRam HR, HORIBA JOBIN YVON). The hydrodynamic diameter of nanoparticles was measured by dynamic light scattering (DLS, Nano ZS90, Malvern).

### Synthesis and Characterization of PAM/BA‐Ag@PDA Hydrogel‐Based Sensors

Six millimeters of 1.0 mg mL^−1^ Ag@PDA NPs and 8.4 mg N,N′‐bis(acryloyl)cystamine (BA, Macklin, N836631) were reacted under 15 min ultrasonication (240 W, 40 KHz). Next, 0.15 g of acrylamide (AM, Sigma, A8887) and 0.01 g of ammonium persulfate (APS, Sigma, 248 614) were dissolved into 1.0 mL of above BA‐Ag@PDA solution. After removing O_2_ with nitrogen flow, 1.0 mL of the PAM/BA‐Ag@PDA precursor was added into a PDMS mold (20 mm × 7 mm × 2 mm) and crosslinked at 60 °C for 12 min. For comparation, the PAM/BA‐Ag@PDA based sensors with different contents of nanoparticles (0.5, 1.0, and 2.0 mg mL^−1^) were synthesized by same procedure. The microstructure and elemental distribution of PAM/BA‐Ag@PDA sensors were analyzed by SEM and EDS mapping. The tensile properties of PAM/BA‐Ag@PDA sensors with dumbbell shape of 20 mm × 7 mm × 2 mm were analyzed by a universal testing machine (ESM303, MARK‐10, USA) with stretch rate of 60 mm min^−1^. The compressive properties of PAM/BA‐Ag@PDA sensors with cylindrical shape of 10 mm × 8 mm were investigated at 90% strain. The conductivity of hydrogels was analyzed by a four‐probe resistivity meter (Helpass, HPS2663).

### Photo‐Thermal, Self‐Healing, and Adhesive Properties of PAM/BA‐Ag@PDA Sensors

The photothermal properties of PAM/BA‐Ag@PDA sensors were detected by a digital thermometer under 808 nm NIR irradiation (0.5, 1.0, 1.5, and 2.0 W cm^−2^) for 5 min. For the thermal stability, the temperature of PAM/BA‐Ag@PDA sensors containing 1.0 mg mL^−1^ of nanoparticles was recorded after four rounds of on/off NIR exposure (1.5 W cm^−2^, 5 min on and 10 min off). For the self‐healing properties, the PAM/BA‐Ag@PDA sensors with or without BA‐Ag@PDA were cut into two pieces, followed by NIR irradiation (2.0 W cm^−2^ at a distance of 10 cm away with spot area of 10 mm diameter) and the healing efficiency was investigated by the tensile test. For tissue adhesion, 300 µL of PAM/BA‐Ag@PDA was added between two pieces of the fresh porcine skin (50 mm × 25 mm × 2 mm), and the lap‐shear test of PAM/BA‐Ag@PDA sensor on porcine skin was measured by the universal testing machine with strain rate of 60 mm min^−1^ under 25 N tensile loading. The lap‐shear strength was determined at the point of detachment.

### Antioxidant Evaluation

The free radical scavenging effect of PAM/BA‐Ag@PDA sensors was investigated using 2,2‐diphenyl‐1‐picrylhydrazyl (DPPH, Alfa Aesar, 44 150) assay. Briefly, 3.0 mL of 100 µm DPPH (in ethanol) was incubated with different concentrations of PAM/BA‐Ag@PDA (0, 5, 10, 15, 20, and 25 mg mL^−1^) at 37 °C in dark for 8 h. Then, the UV absorption of DPPH was measured at 517 nm. The radical scavenging efficiency was calculated by the following equation: Scavenging efficiency = (1‐A_h_/A_0_) × 100%. A_0_ and A_h_ were the A_517_ values of DPPH before and after incubation, respectively.

### Antibacterial Activity Evaluation

The in vitro antibacterial efficiency of PAM/BA‐Ag@PDA sensor was measured against Escherichia coli (*E. coli*, ATCC25922) and *Staphylococcus aureus* (*S. aureus*, ATCC29213) via plate count assay. 10 µL of bacterial suspension (1 × 10^8^ CFUs mL^−1^) was incubated with 150 µL of 1.0 mg mL^−1^ PAM/BA‐Ag@PDA sensor with or without NIR irradiation (1.5 W cm^−2^, 3 min) in a 48‐well plate. 10 µL of bacterial suspension (1 × 10^8^ CFUs mL^−1^) in 150 µL of PBS was set as control. After incubation at 37 °C for 2 h, 1.0 mL of fresh PBS was supplemented into above bacteria suspensions, of which 100 µL of suspension was transferred onto the nutrient agar plate and incubated at 37 °C for another 12 h. For the in vivo infection model, male SD rats (4 weeks, Guangdong Vital River Laboratory Animal Technology Co., Ltd.) were anesthetized by isoflurane (R510‐22, RWD) and divided into three groups (*n* = 3). Briefly, the dorsal region of rat was shaved, where a Φ10 mm burn wound was created using a pre‐heated (100 °C) aluminum bar (49.2 g) for 20 s. After 6 h of burn wound treatment, the necrotic tissue was removed, and a fresh full‐thickness cutaneous wound was generated by a Φ10 mm biopsy punch. Next, the wound was injected with 10 µL of S. aureus suspension (1 × 10^8^ CFUs mL^−1^) and treated by following groups: PBS, PAM/BA‐Ag@PDA or PAM/BA‐Ag@PDA+NIR (1.5 W cm^−2^, 3 min). After 1 day of treatment, samples in each group were collected, resuspended with PBS, and incubation on the agar plates for another 18 h at 37 °C. Then, the bacterial survival ratio of each group was also evaluated by plate count assay. Animal experiments were evaluated and approved by the Institutional Animal Care and Use Committee (YSB‐20220316‐TW‐A0526) of Shenzhen Institute of Advanced Technology, Chinese Academy of Sciences. The colony‐forming units (CFUs) of bacteria could be counted and photographed. The bacterial survival ratio was calculated as follows:

(1) $$\begin{array}{ccc} & & \mathrm{Bacterial}\;\mathrm{survival}\;\mathrm{ratio}\left(\%\right)\\ & & \;=\frac{\mathrm{Survivor}\;\mathrm{bacterial}\;\mathrm{counts}\;\mathrm{of}\;\mathrm{experimental}\;\mathrm{group}}{\mathrm{Bacterial}\;\mathrm{counts}\;\mathrm{of}\;\mathrm{control}}\;\times \;100\% \end{array}$$

### Biocompatibility Evaluation

Cell viability of PAM/BA‐Ag@PDA sensor was performed using Cell Counting Kit‐8 (CCK‐8, Beyotime, C0043) method.^[^ ^59^ ^]^ and Live/Dead staining (Thermo, L3224). 1.0 mL of L929 fibroblast cells (CL‐0137, Procell) at a density of 4 × 10^4^ cells per well were co‐incubation PAM/BA‐Ag@PDA sensor at 37 °C in a 5% CO_2_ humidified incubator for 1, 3, and 5 days. Cells seeded on blank well were set as control. For cell proliferation, the culture medium was replaced by 10% (v/v%, in culture medium) CCK‐8 solution and incubated for 2 h. The optical density (OD) value at 450 nm was measured by a microplate reader (TECAN, Infinite200 PRO). To assess cytotoxicity, cells were co‐stained with calcein acetoxymethyl ester (calcein‐AM) and propodium (PI) following the manufacturer's instructions and imaged under confocal laser‐scanning microscopy (CLSM, LSM880, ZEISS).

### Statistical Analyses

Statistical analysis was performed with GraphPad Prism 9. All experimental data were presented as the mean ± SD. For comparison of two groups, two‐tailed Student's *t* tests were used. For comparison of multiple experimental groups, either one‐way analysis of variance (ANOVA) or two‐way ANOVA method was used where indicated. The *p* value < 0.05 was considered statistically significant. ^*^ *p* < 0.05, ^**^ *p* < 0.01, ^***^ *p* < 0.001, and ^****^ *p* < 0.0001, ns not significant.

## Conflict of Interest

The authors declare no conflict of interest.

## Author Contributions

W.T., H.L., Y.L., and G.L. proposed the concept, supervised the project and finalized the manuscript. W.S. and J.C. executed the experiments and collected the data. C.X., M.L., and J.Z. assisted with the experiments. W.T., W.S., H.L., and J.C. analyzed the data and wrote the manuscript. W.T. was in charge of the logical writing, editing, and figures preparation. Y.C., C.C., and K.C. participated in discussing data interpretation. W.S., H.L., and J.C. contributed equally to this work.

## Supporting information

Supporting Information

## Data Availability

The data that support the findings of this study are available from the corresponding author upon reasonable request.
