# Supplementary material for: Stretchable, Self‐Healing, and Bioactive Hydrogel with High‐Functionality N,N′‐bis(acryloyl)cystamine Dynamically Bonded Ag@polydopamine Crosslinkers for Wearable Sensors
Source: Adv Sci (Weinh). 2024 Jul 19;11(35):2404451. doi: 10.1002/advs.202404451 (PMC11425271; doi:10.1002/advs.202404451)
Supplement: Supplementary file 1 — Supporting Information [file ADVS-11-2404451-s001.docx]

Supporting Information

Stretchable, self-healing and bioactive hydrogel with high-functionality N,N’-bis(acryloyl)cystamine dynamically bonded Ag@polydopamine crosslinkers for wearable sensors

*Wei Shi^1^, Hui Li^1^, Jing Chen^1^, Yern Chee Ching*, Cheng Hock Chuah, Chengsheng Xu, Moran Liu, Jinyong Zhang, Kuan Yong Ching, Yongsheng Liang*, Guanglin Li*, Wei Tang**


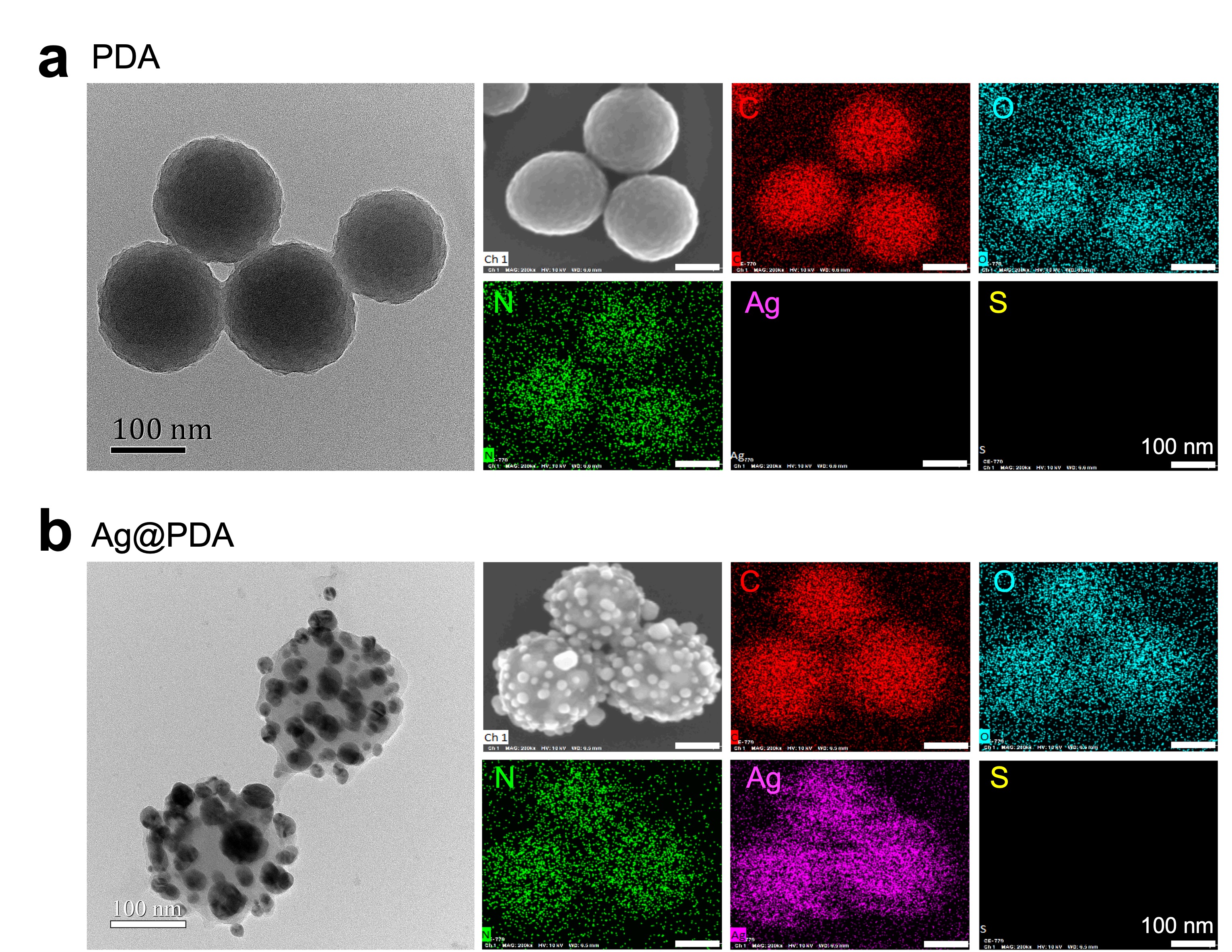


**Figure S1.** TEM, SEM and EDS mapping images of the a) PDA nanoparticles and b) Ag@PDA nanoparticles.


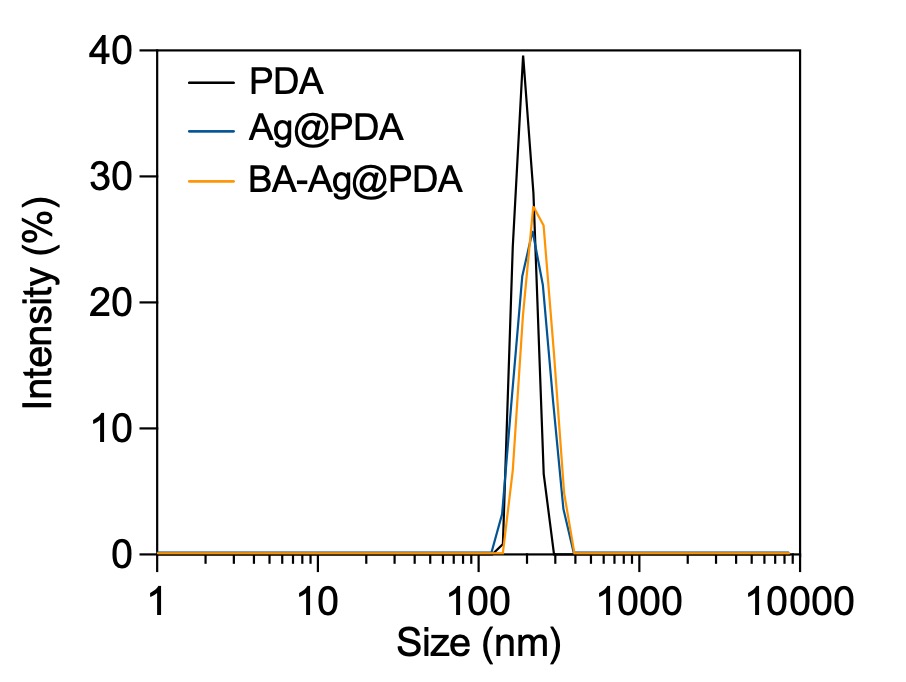


**Figure S2.** Size distribution of the PDA, Ag@PDA and BA-Ag@PDA nanoparticles.


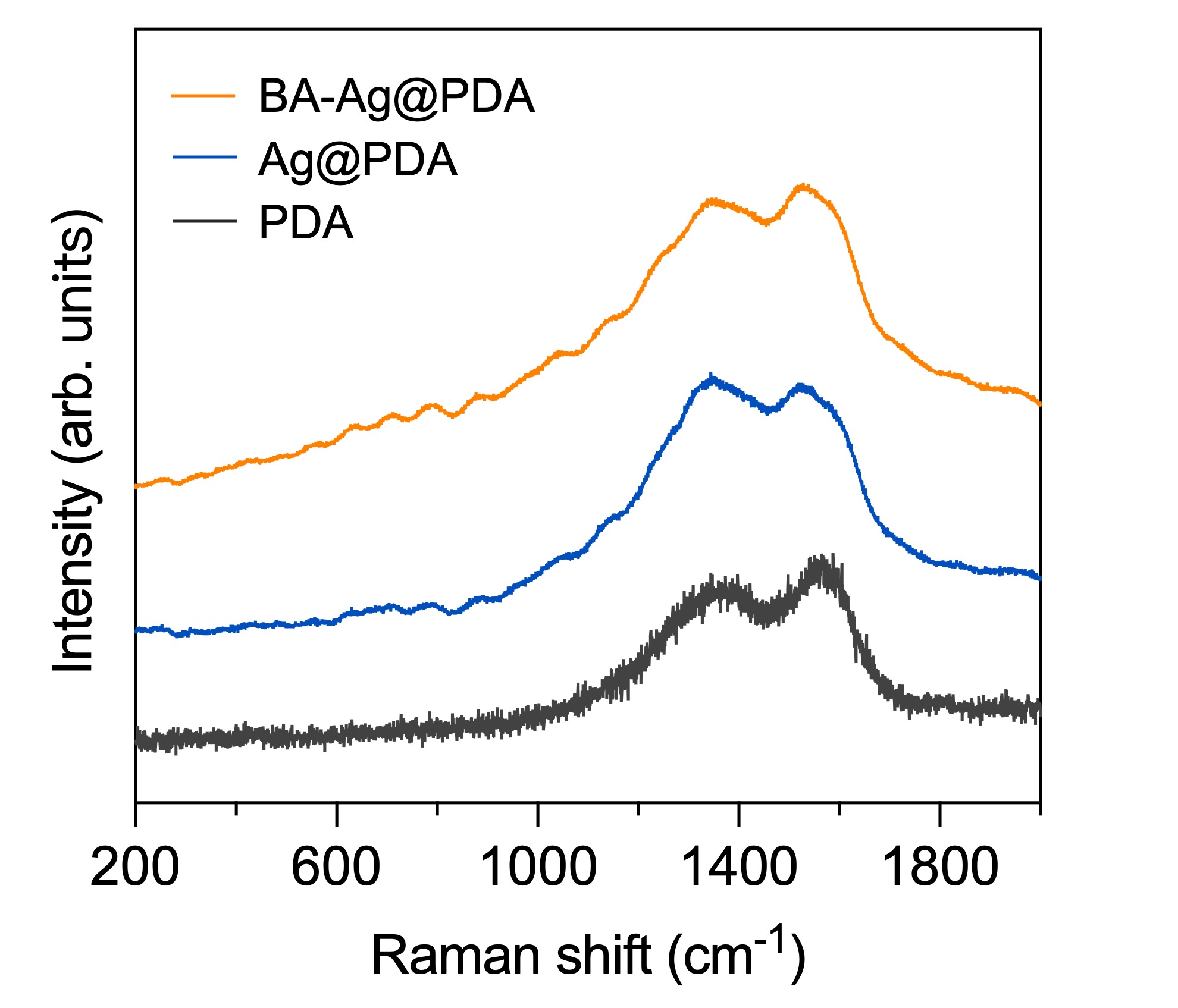


**Figure S3.** Raman curves of the PDA, Ag@PDA and BA-Ag@PDA nanoparticles.


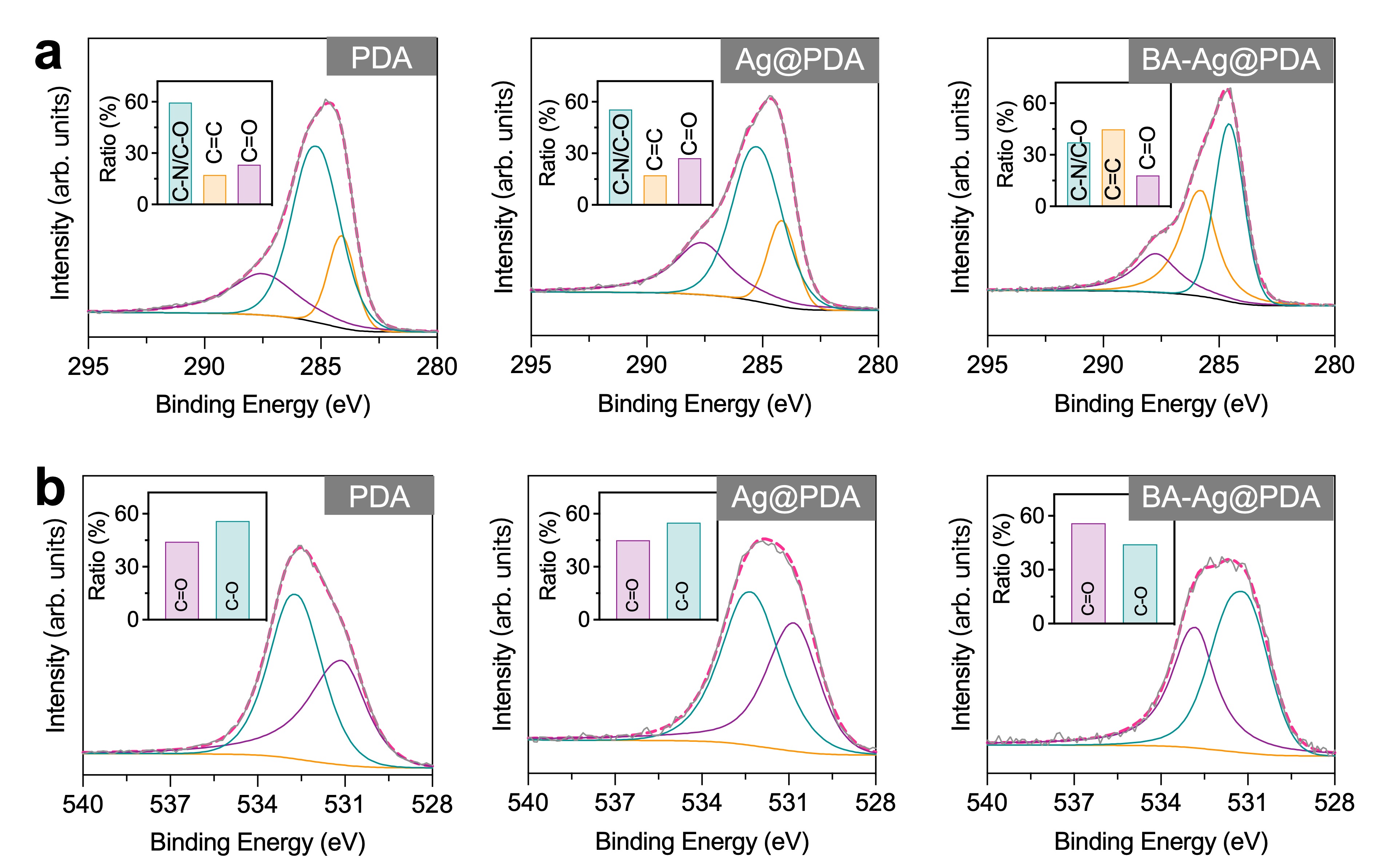


**Figure S4.** XPS spectra of the a) C1s and b) O1s of the PDA, Ag@PDA and BA-Ag@PDA nanoparticles. The peaks in the C1s spectra at 284.1, 285.16 and 287.5 eV assigned to C=C, C-N/C-O and C=O bonds, respectively, were observed in all groups due to the organic composition of PDA. There was no obvious difference between PDA and Ag@PDA, either in C1s or O1s spectra. Comparatively, the highest levels of C=C (44.84%) and C=O (55.87%) were found in the C1s and O1s spectra of BA-Ag@PDA, respectively. The increased percentage of C=C and C=O suggested the successful modification of Ag@PDA with BA.


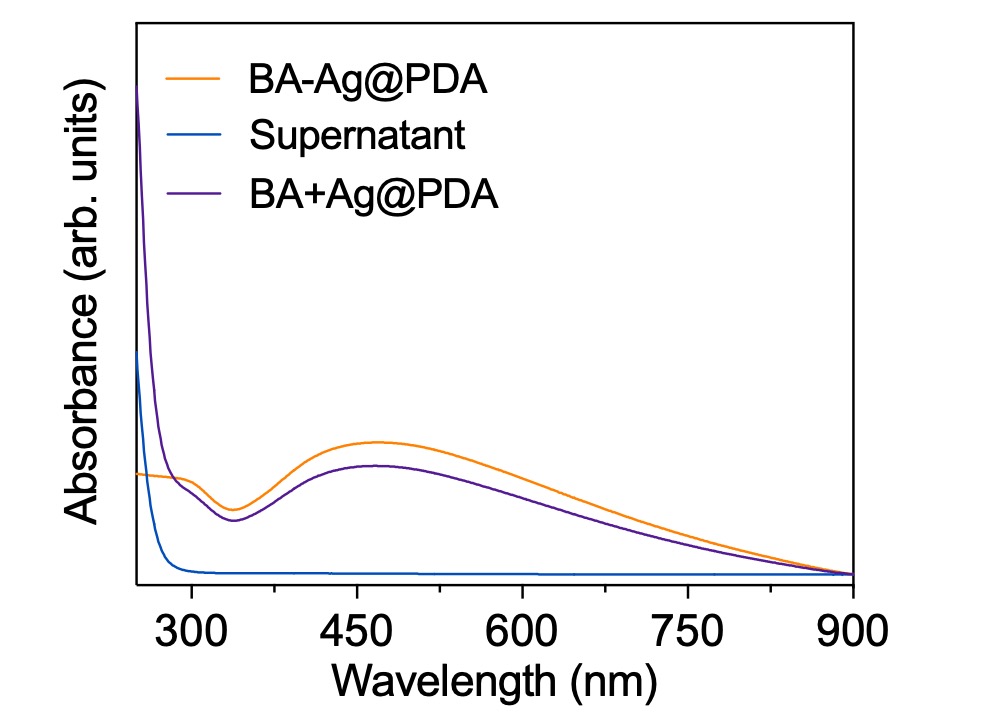


**Figure S5.** UV-vis-NIR absorption spectra of the BA-Ag@PDA before and after reaction.


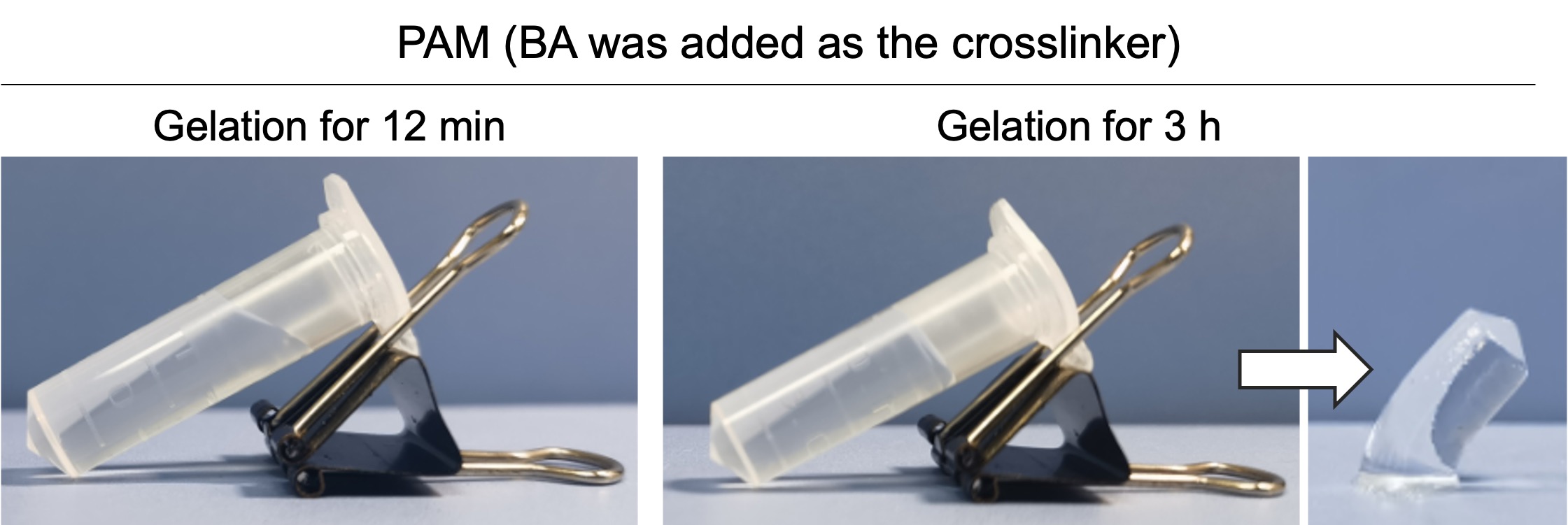


**Figure S6.** Gelation behavior of the PAM hydrogel using BA as the crosslinker.


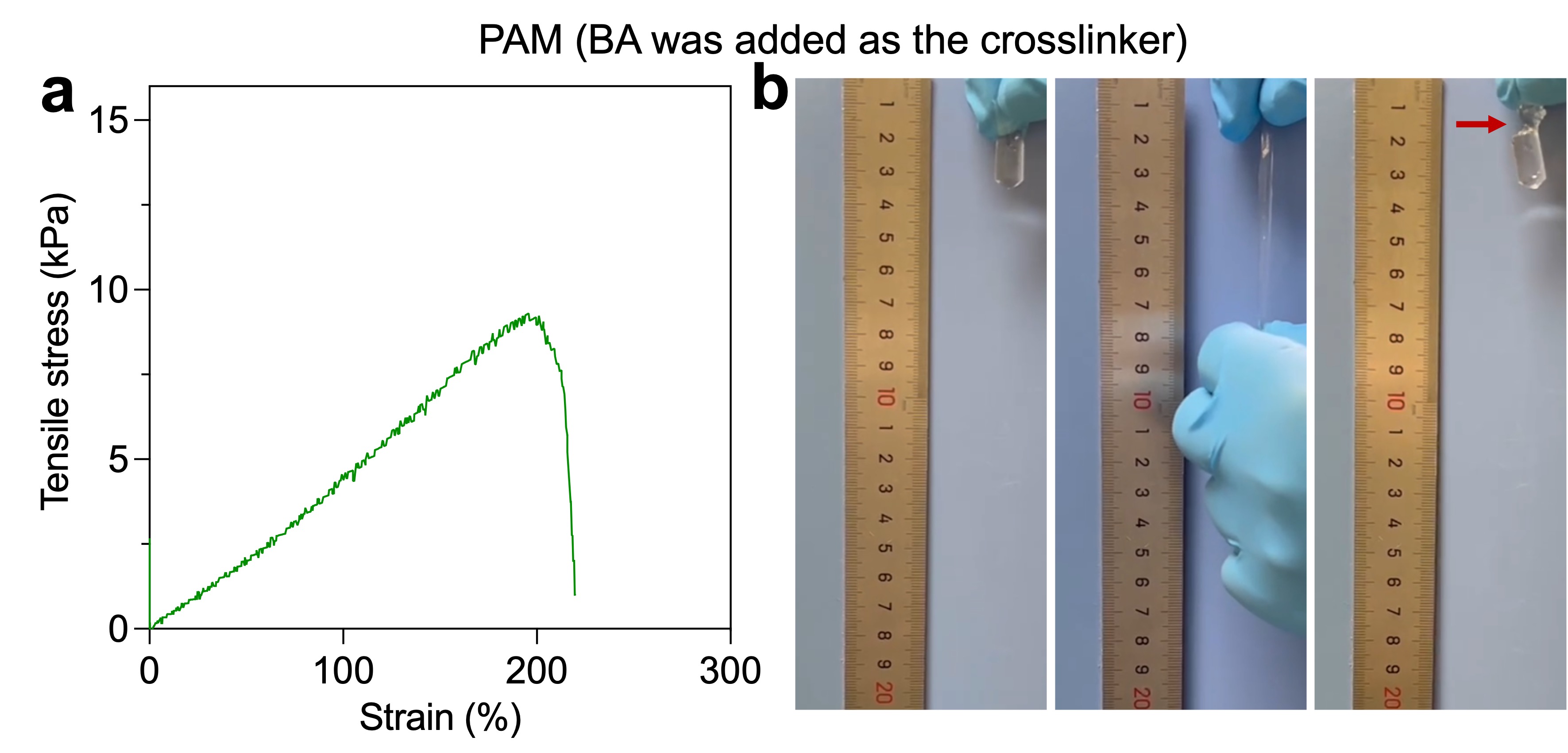


**Figure S7.** (a) Tensile stress-strain curve of the PAM hydrogel. (b) Photos of the PAM hydrogel before and after stretching.


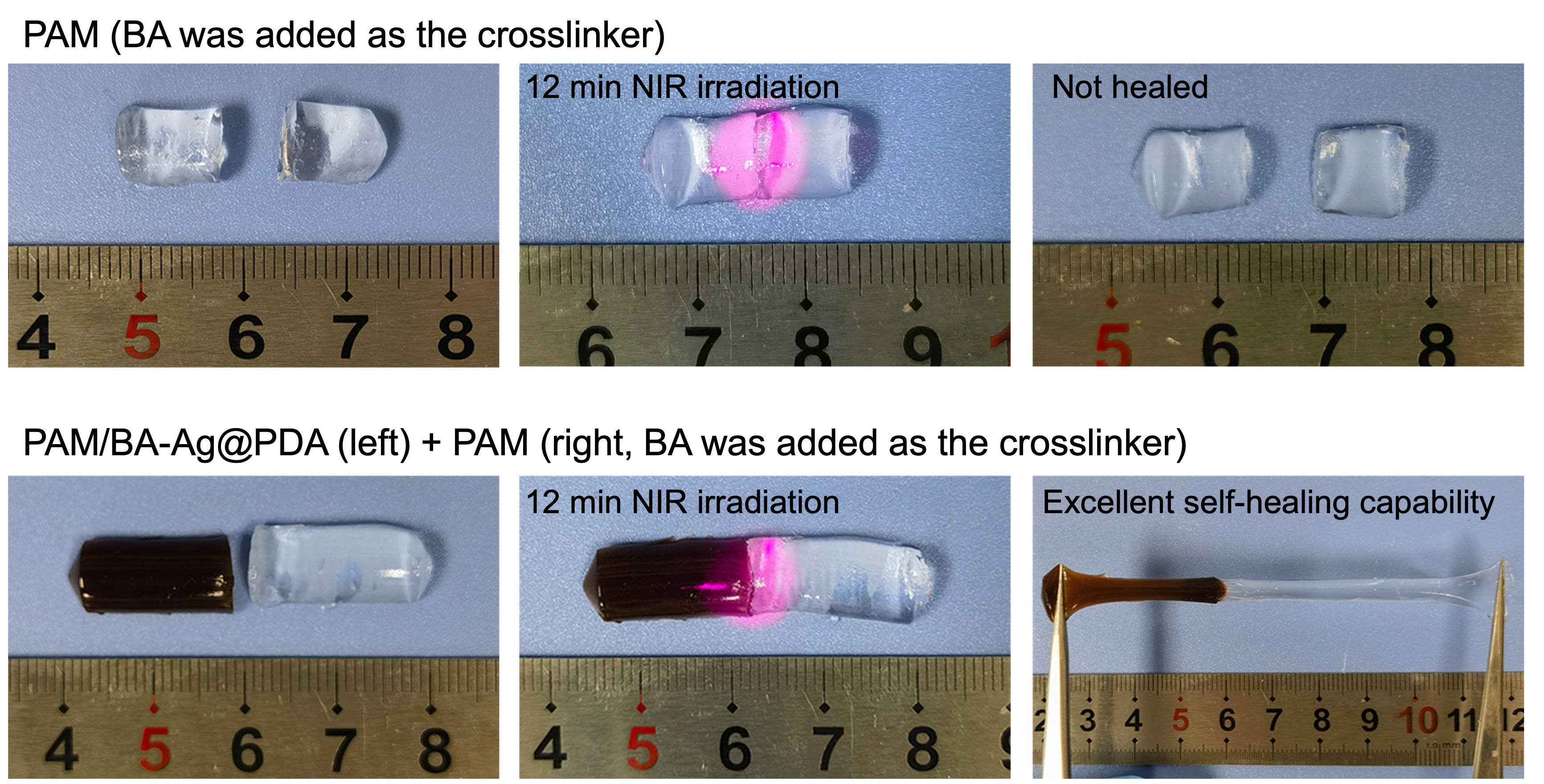


**Figure S8.** Self-healing behavior of the PAM and PAM/BA-Ag@PDA hydrogels under NIR irradiation.


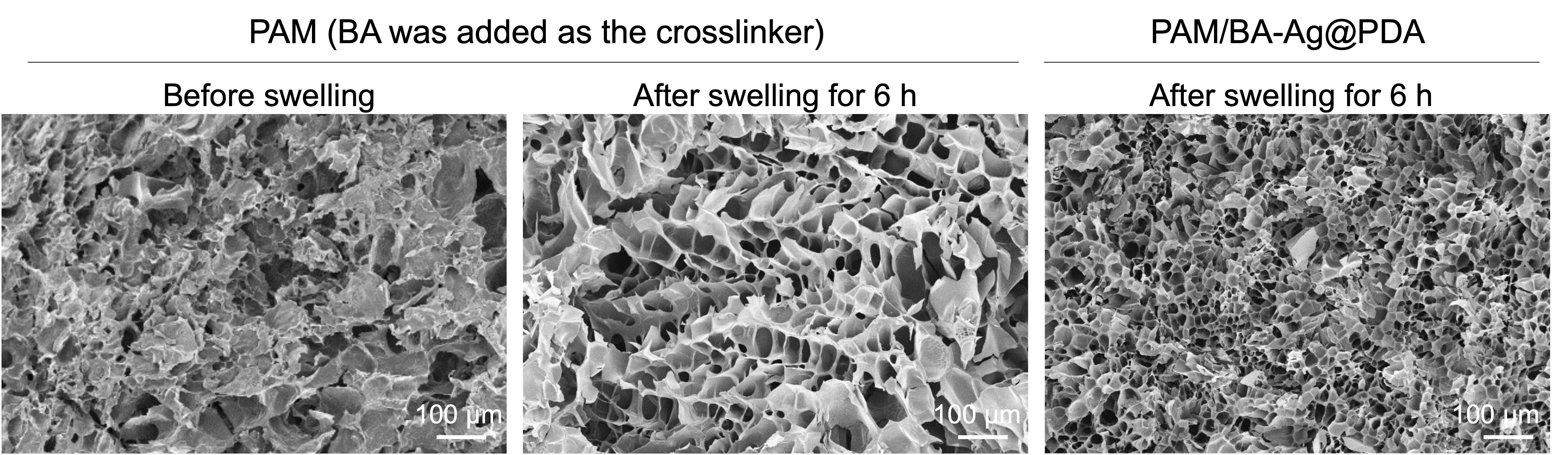


**Figure S9.** SEM images of the hydrogel networks.


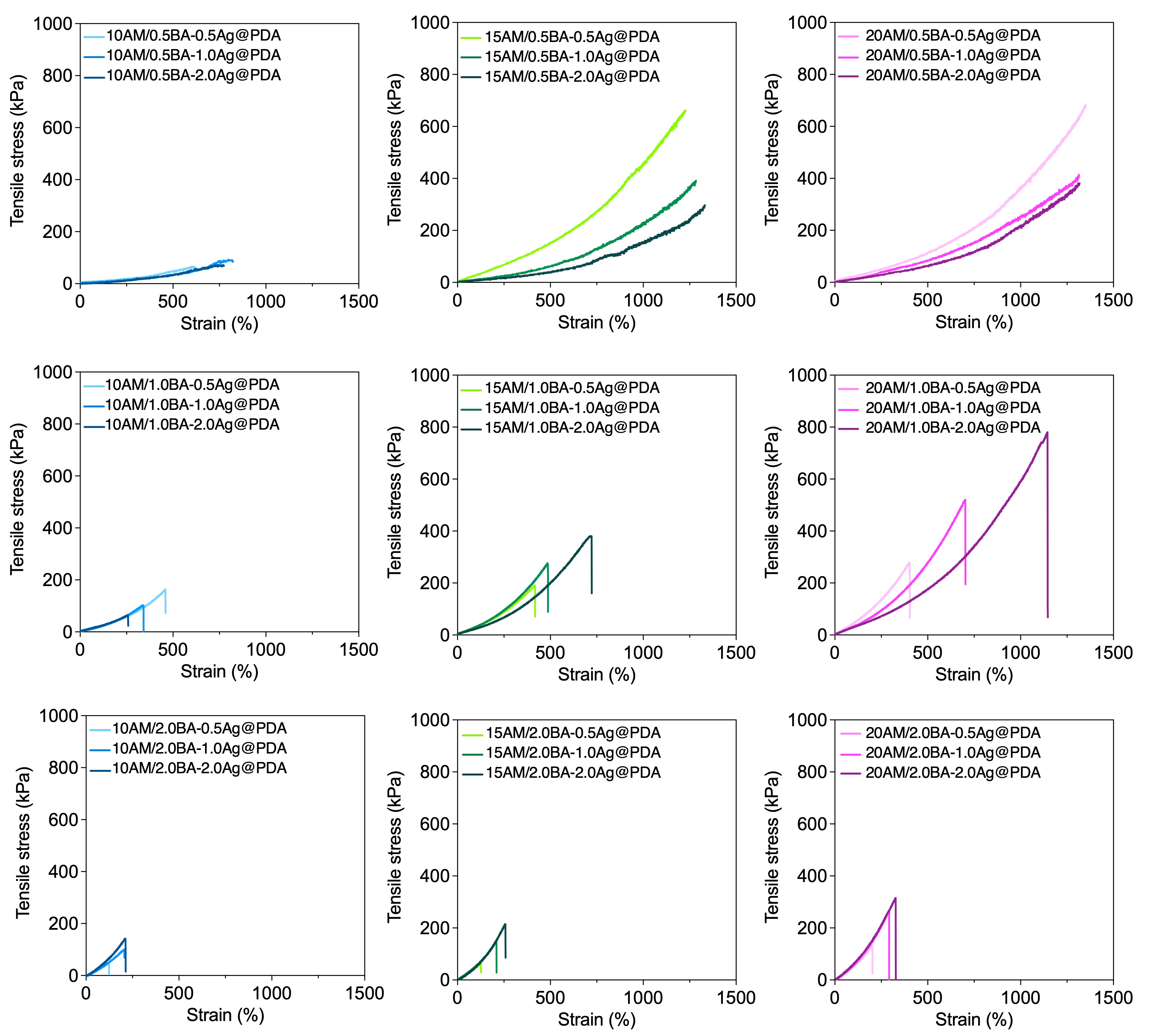


**Figure S10.** Tensile stress-strain curves of the PAM/BA-Ag@PDA hydrogels with different contents of AM, BA and Ag@PDA.


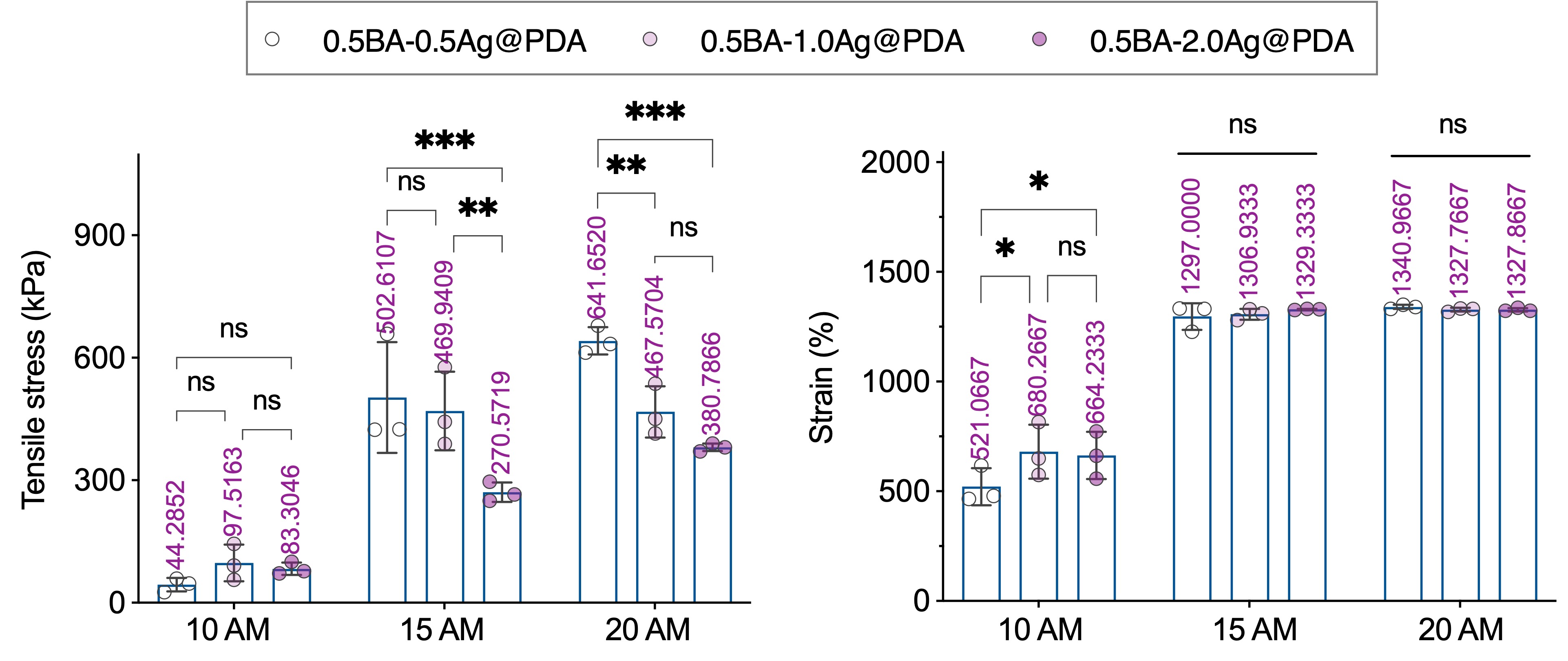


**Figure S11.** Tensile stress and tensile strain of the PAM/BA-Ag@PDA hydrogels with different contents of AM and Ag@PDA. The BA content was kept at 0.5 mg mL^-1^.


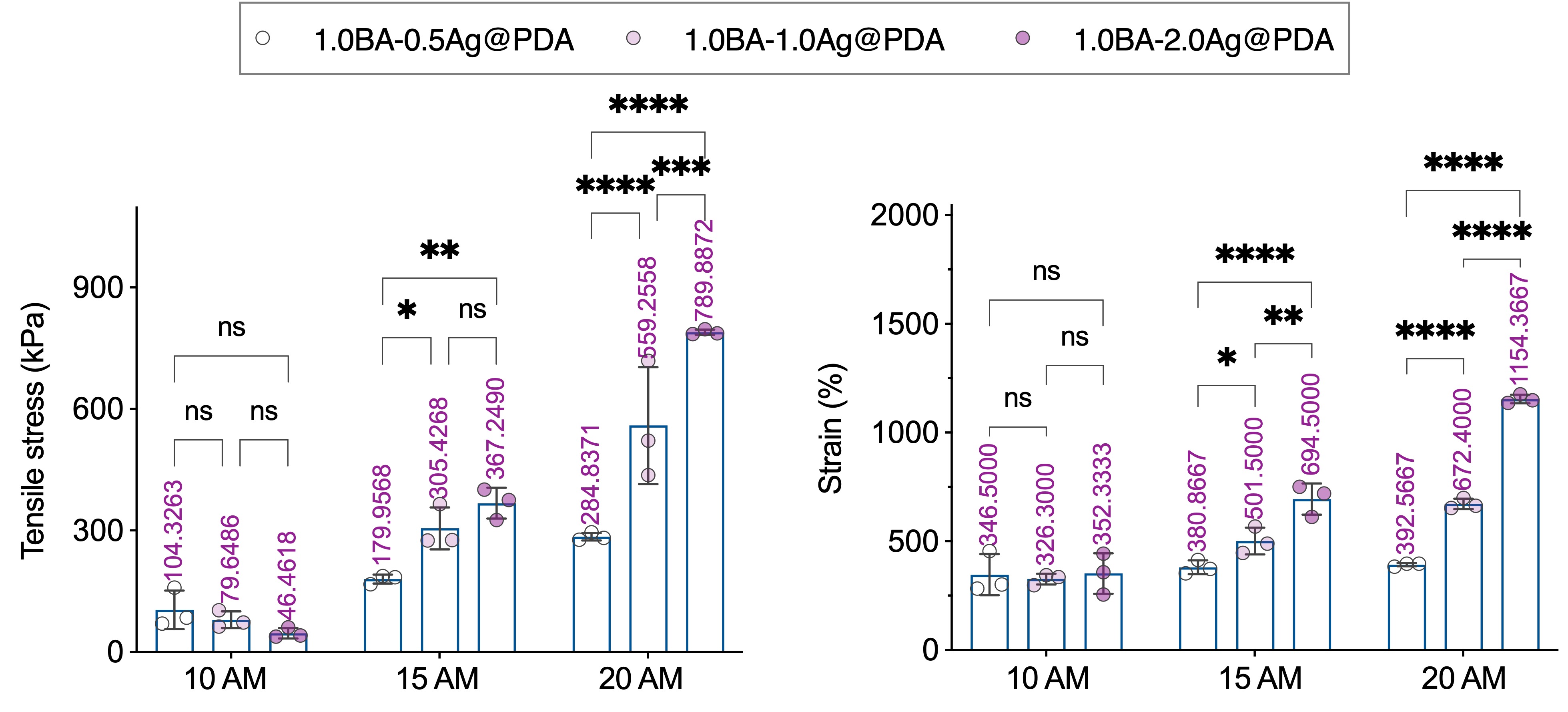


**Figure S12.** Tensile stress and tensile strain of the PAM/BA-Ag@PDA hydrogels with different contents of AM and Ag@PDA. The BA content was kept at 1.0 mg mL^-1^.


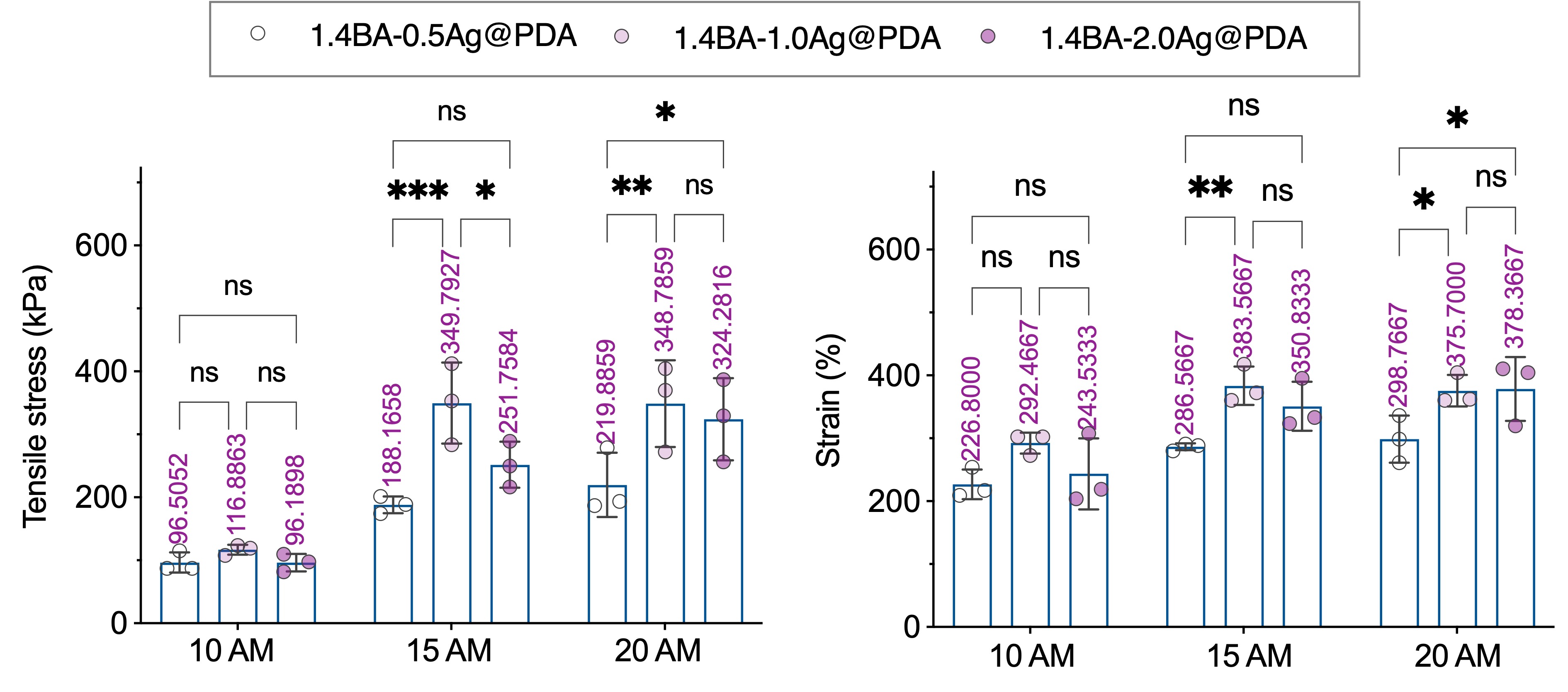


**Figure S13.** Tensile stress and tensile strain of the PAM/BA-Ag@PDA hydrogels with different contents of AM and Ag@PDA. The BA content was kept at 1.4 mg mL^-1^.


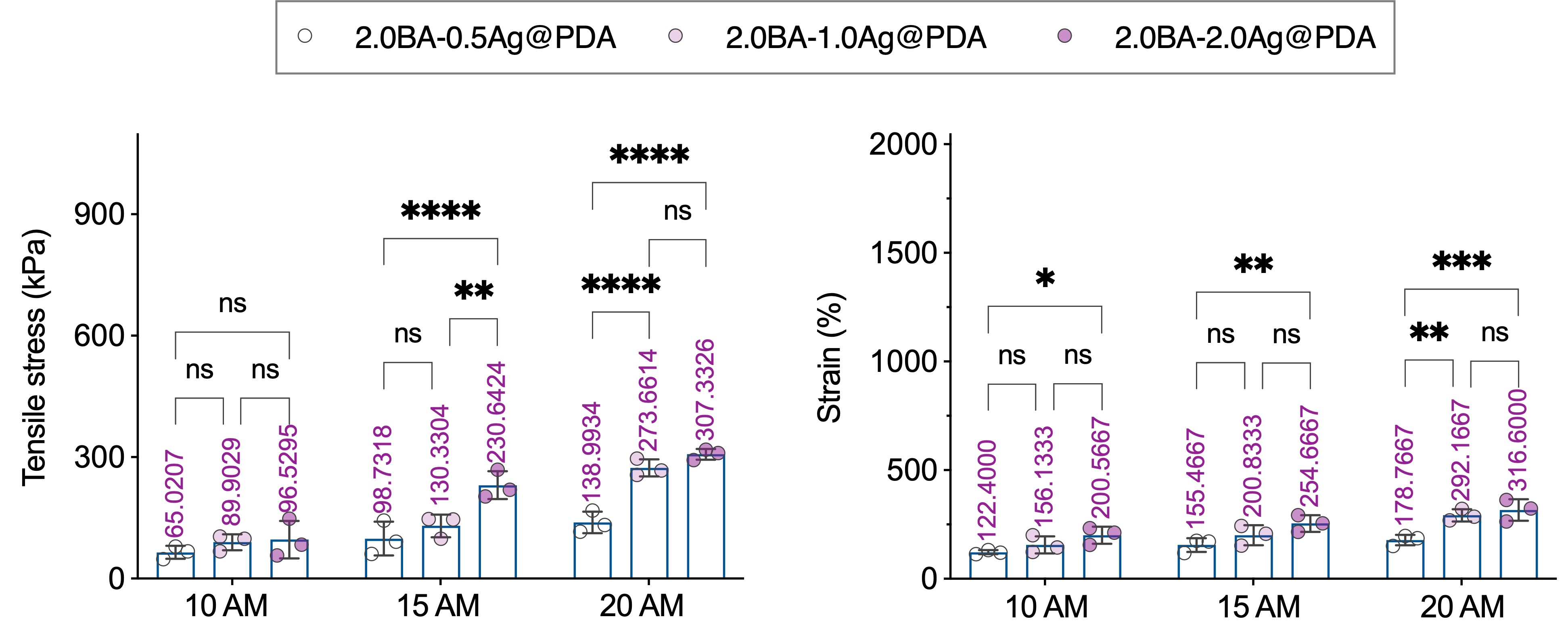


**Figure S14.** Tensile stress and tensile strain of the PAM/BA-Ag@PDA hydrogels with different contents of AM and Ag@PDA. The BA content was kept at 2.0 mg mL^-1^.


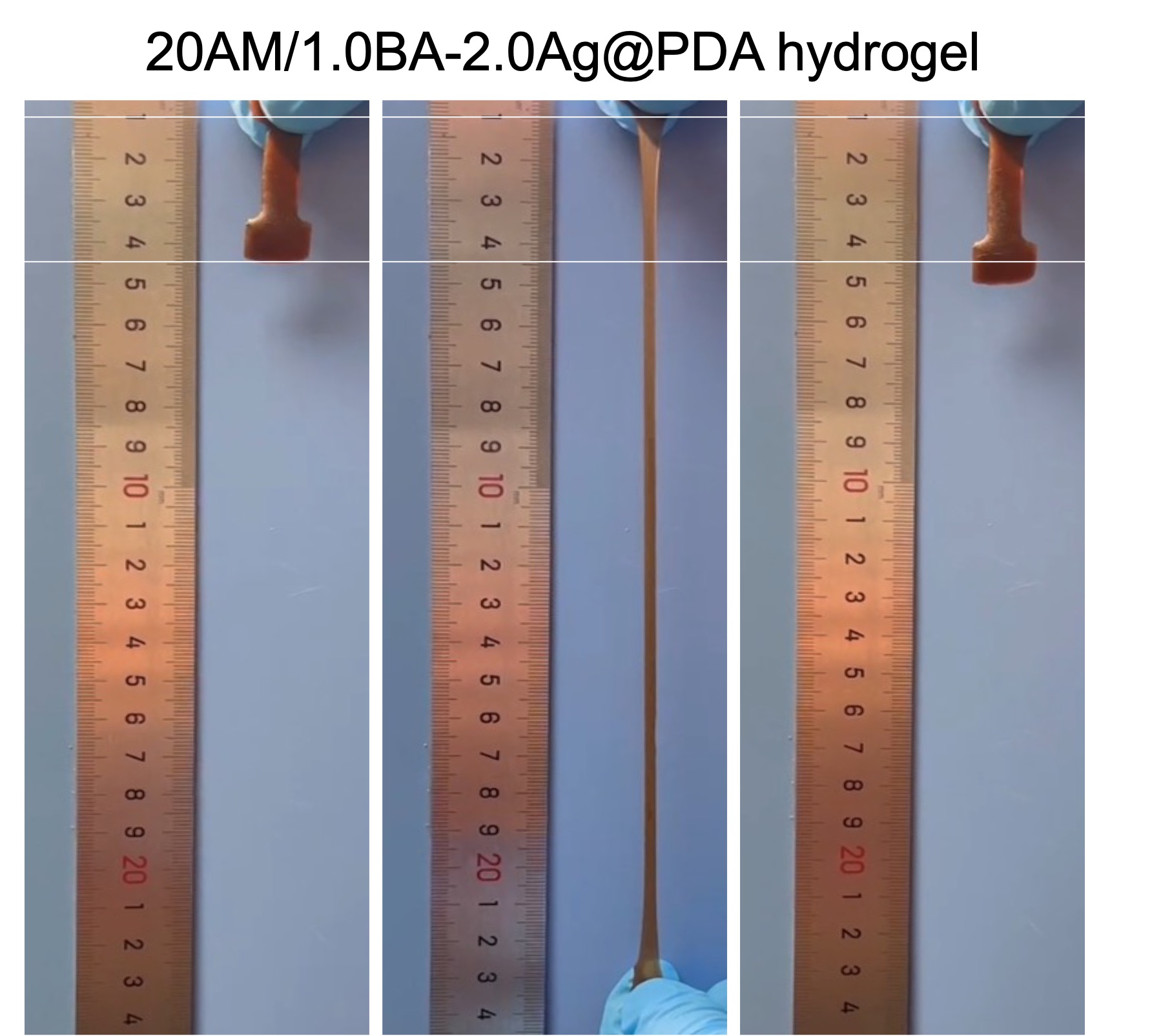


**Figure S15.** Images of the 20AM/1.0BA-2.0Ag@PDA hydrogel before and after stretching.


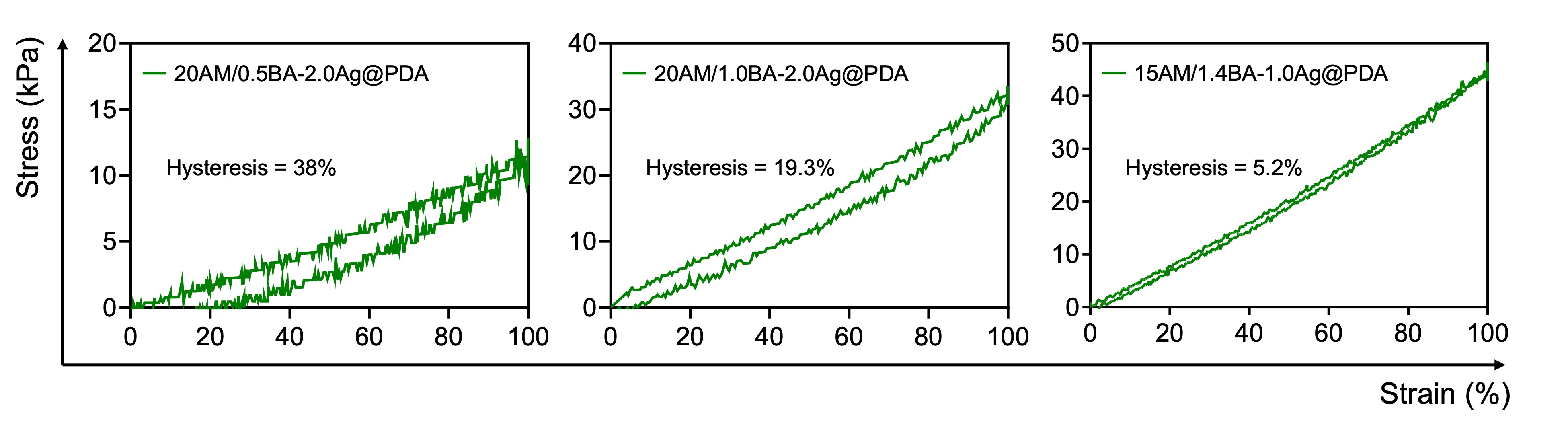


**Figure S16.** Hysteresis curves of the PAM/BA-Ag@PDA hydrogels with different contents of AM, BA and Ag@PDA.


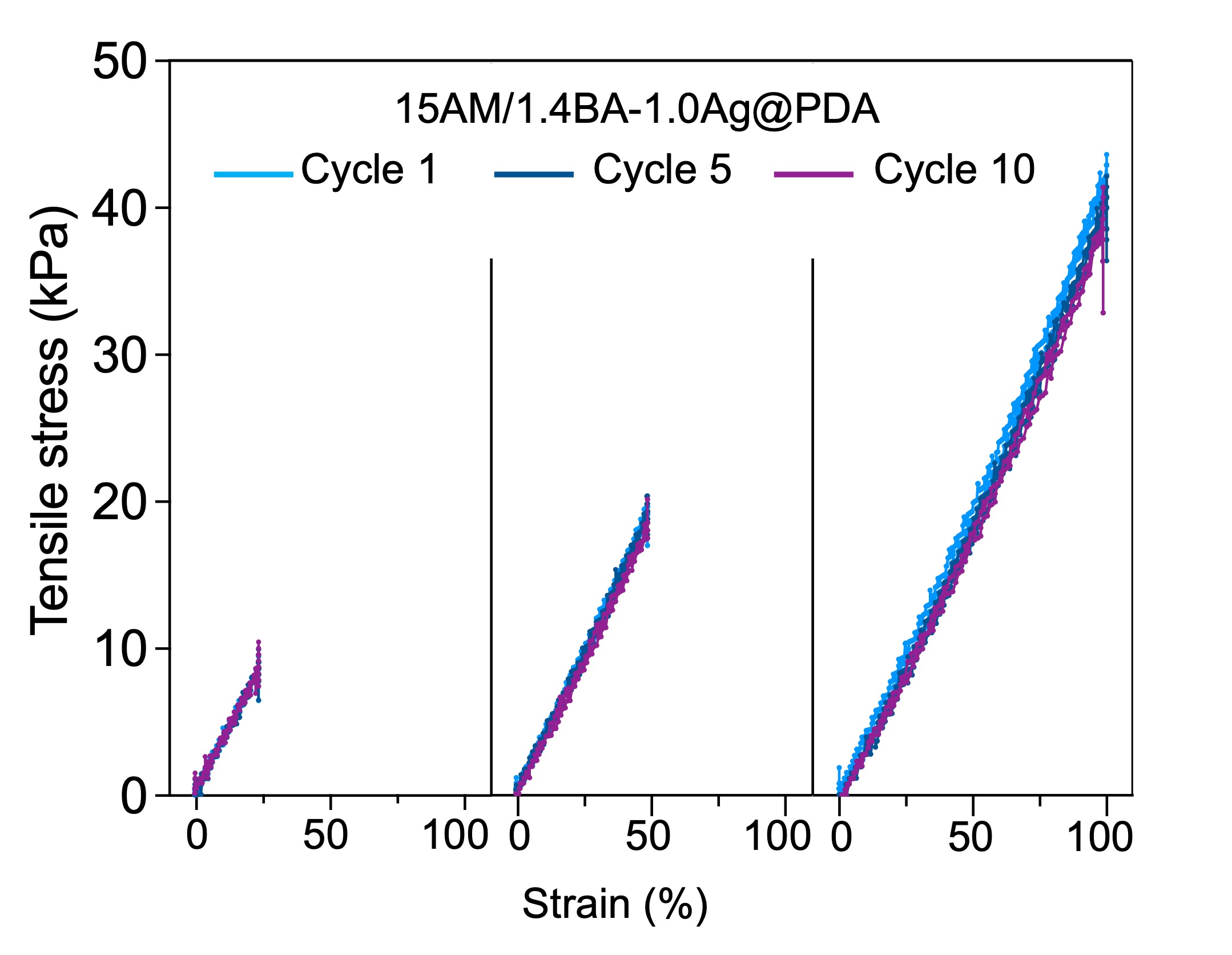


**Figure S17.** The tensile stress-strain curves of the 15AM/1.4BA-1.0Ag@PDA hydrogels during 10 loading-unloading cycles at a maximum strain of 25%, 50%, and 100%.


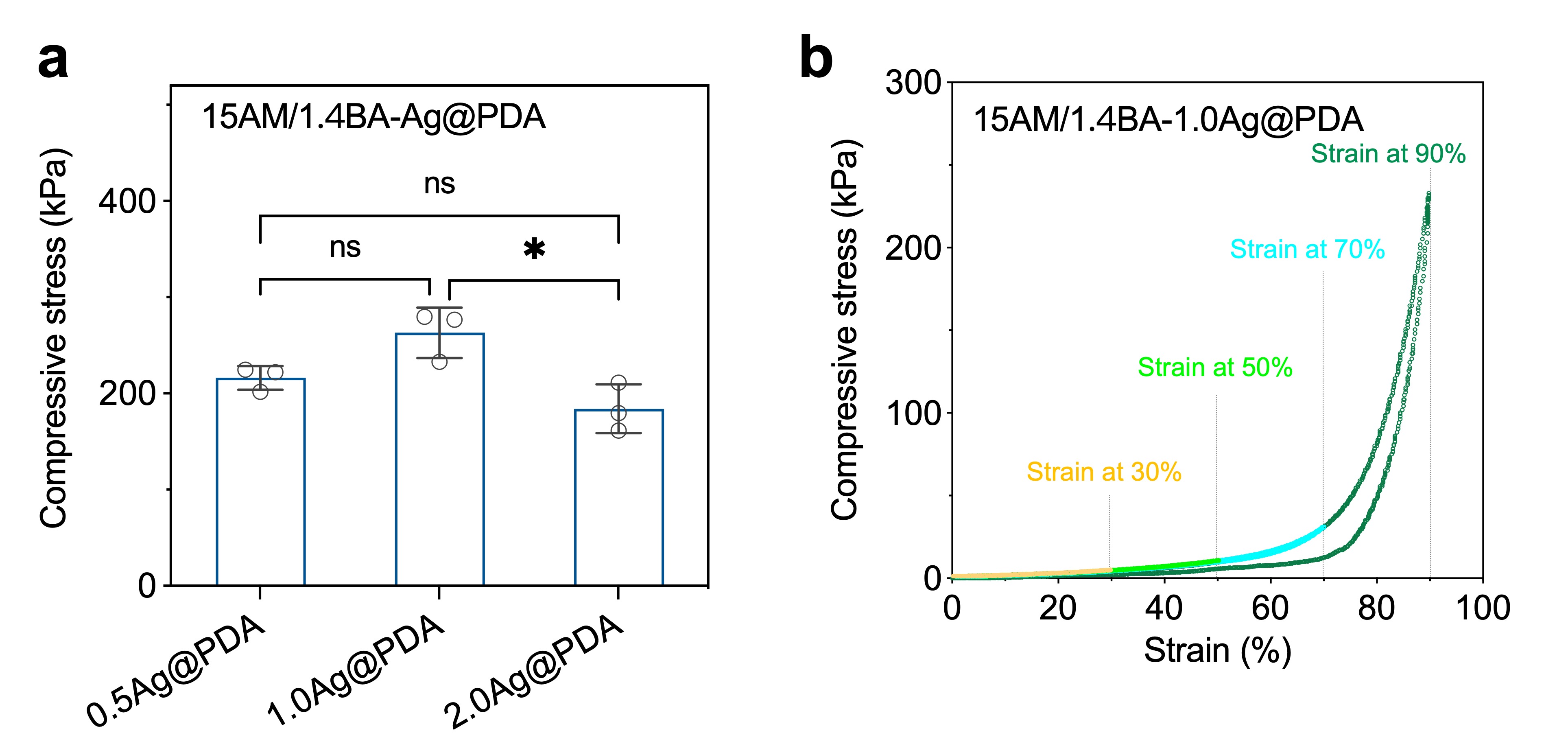


**Figure S18.** a) The compressive strength of the PAM/BA-Ag@PDA hydrogels with different Ag@PDA contents. b) The stress resilience of the PAM/BA-Ag@PDA hydrogels at 30%, 50%, 70% and 90% strain.


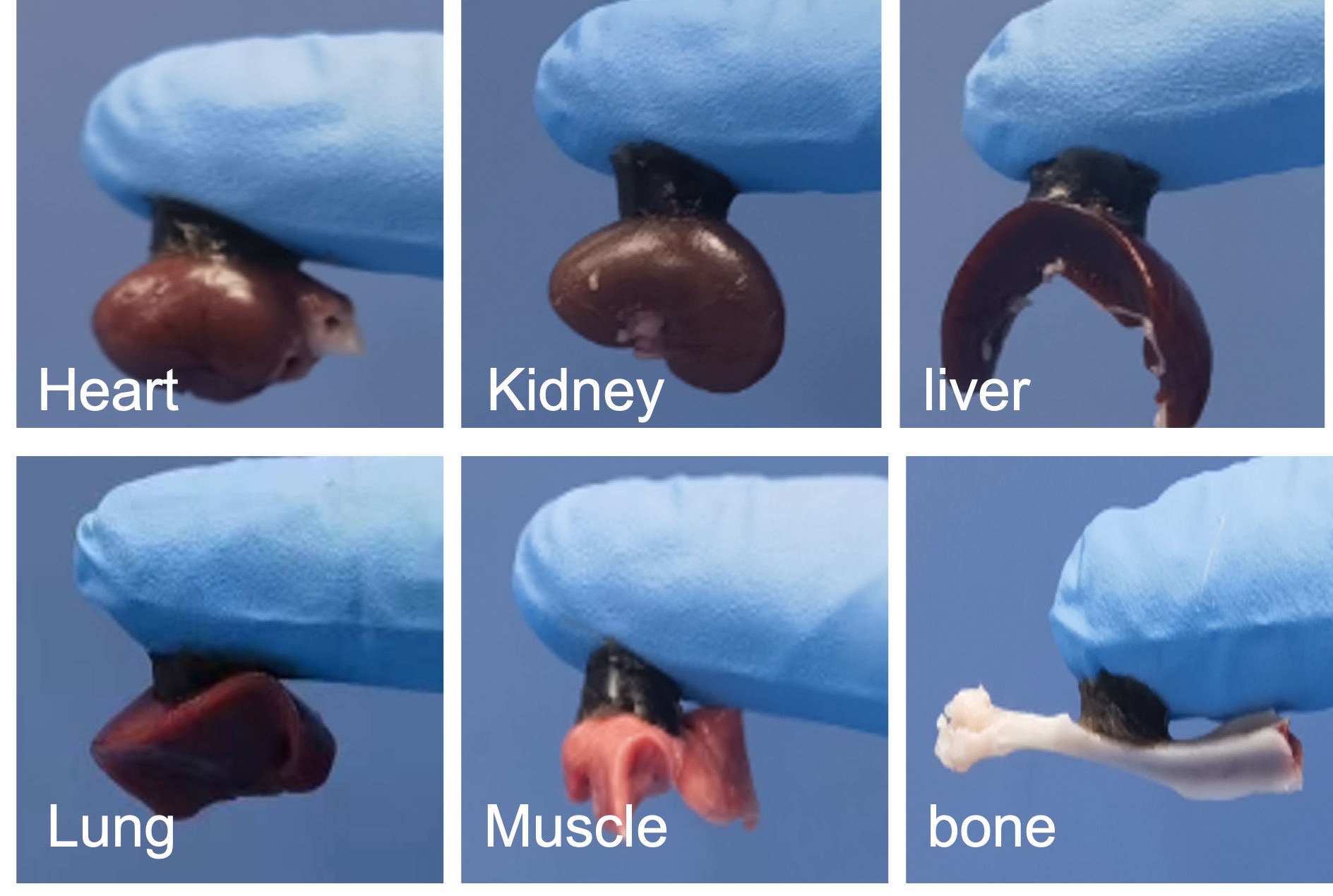


**Figure S19.** The adhesion properties of the 15AM/1.4BA-1.0Ag@PDA hydrogels on rat fresh organs or tissues.


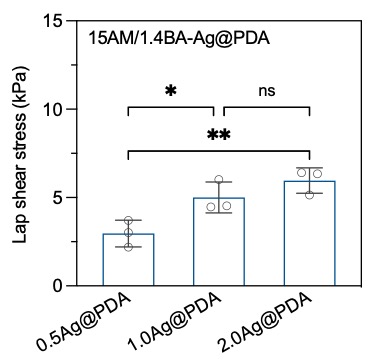


**Figure S20.** The lap-shear strength of the PAM/BA-Ag@PDA hydrogels with different Ag@PDA contents.


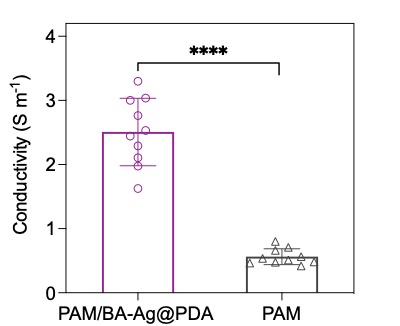


**Figure S21.** Conductivity of the PAM/BA-Ag@PDA and PAM (BA was added as the crosslinker) hydrogels.


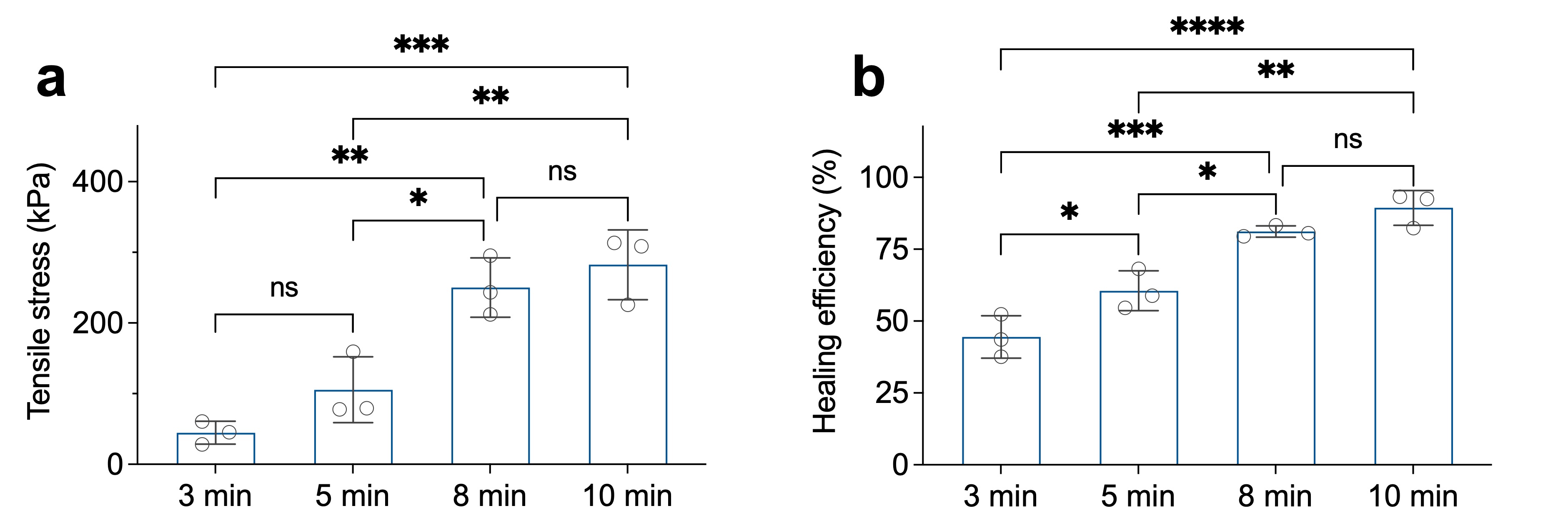


**Figure S22.** a) The tensile stress, b) Healing efficiency of the self-healed PAM/BA-Ag@PDA sensors after 3, 5, 8 and 10 min NIR exposure.

**
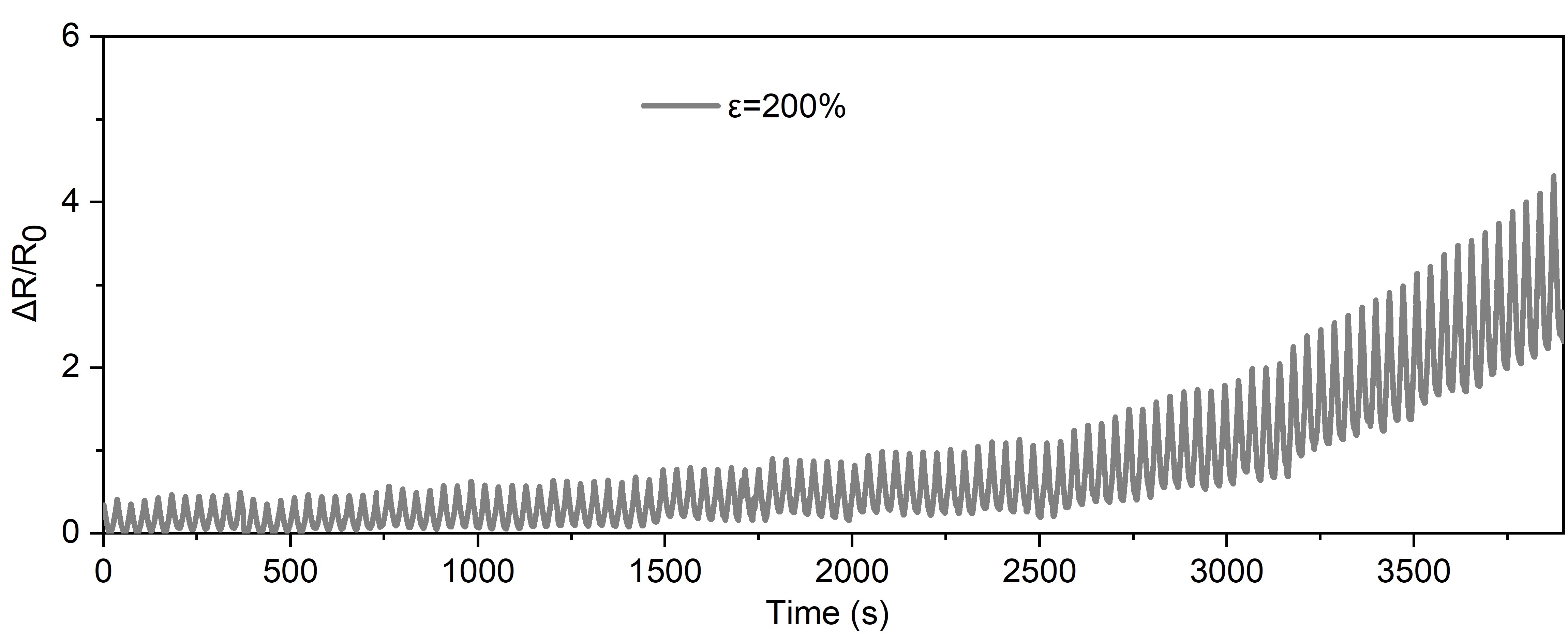
**

**Figure S23.** Relative resistance changes of the healed PAM/BA-Ag@PDA hydrogel sensor at 200% strain for 100 consecutive cycles.

**Table S1** Mechanical, electrical, and biological properties of wearable hydrogel sensors.

| Materials | Tensile  stress/strain | Compressive stress/strain | Conductivity (S m^-1^) | Gauge factor  (strain range) | Response  time | Healing  time | Anti-bacterial  (Y/N) | Anti-  oxidative  (Y/N) | Ref. |
| --- | --- | --- | --- | --- | --- | --- | --- | --- | --- |
| 15AM/1.4BA-1.0Ag@PDA | 349.80 kPa 383.57% | 263.08 kPa 90% | 2.51 | 1.86 | 138 ms | 10 min | Y | Y | This  work |
| PAM/gelatin/  CNT-TA-Ag | <0.45 MPa  <300% | N | 0.11 | 1.87 | 206 ms | 2 min | Y | N | [1] |
| PAM/  PDA@CNT | 17.5 kPa  >700% | N | 0.002 | 1.99 (0-200%)  3.93 (200-400%) | 76 ms | 6 h | N | N | [2] |
| AA/diacetone acrylamide | 88.7 kPa  1300% | N | - | 0.871(0-100%)  1.28 (100-200%)  1.71 (200-400%) | 115 ms | 24 h | N | N | [3] |
| PAA/GPC | <0.4 MPa  1180% | N | 0.0004 | 2.95  at small strain | 230 ms | 6 h | N | N | [4] |
| Alginate/PVA  /PEDOT:PSS | 39.8 kPa  1200% | 242 kPa  80% | 3.61 | 0.90 (0-340%)  1.50 (340-680%)  1.86 (680-1000%) | 830 ms | 10 min | N | N | [5] |
| Acrylic acid  (AA)/Fe^3+^ | 146.6 kPa  1708.7% | N | 1.22 | 0.83 (0-100%)  2.54 (100-600%)  3.93 (600-1500%) | N | 1 h | N | N | [6] |
| PVA-CA-Mxene | 9.7 kPa  1100% | N | - | 2.3 (200%) | N | 0.12 s | N | N | [7] |
| PVA/AAc/NaCl | 400 kPa  550% | <100 kPa  45% | 0.25 | 1.21 (0-100%)  2.29 (100-300%)  3.25 (>300%) | N | 12 h | N | N | [8] |
| ZCS-MA/GSH | N | 28.7 kPa  50% | 0.35 | 0.3 (10%)  2.8 (10-40%)  11.6 (40-60%) | 140 ms | 1 h | N | N | [9] |
| PAAm-Clay-T | 110 kPa  4052% | N | 9.95 | 1.86 (0-200%) | N | 2 h | N | N | [10] |
| PVA/PEG/TA-Mxene | 930 kPa  400% | 510 kPa  50% | 8.1 | 1.12 | 60 ms | - | N | N | [11] |
| SF/TA@PPy | <4 kPa  >500% | N | 0.034 | 0.7154 | N | 30 s | Y | N | [12] |
| Agar/Borax  /MXene | 129 kPa  105.1% | N | 8.14 | 1.44 | N | 180 min | N | N | [13] |
| PAM/PBA-IL/CNF | 369.5 kPa  1810% | N | 0.56 | 3.41 (0-300%)  8.36 (300-1000%) | 195 ms | 150 min | N | N | [14] |
| PANI/  P(PEG-co-AA) | 25.9 kPa  580% | <135.8 kPa  90% | 7.432 | 1.46 (0-50%), 2.43 (50-200%) | 140 ms | 10 min | N | N | [15] |
| DMP/IL/PVA | 130 kPa  <250% | 25 kPa  65% | 0.96 | 1.2 (0-50%), 1.8 (50-200%) | 117 ms | - | N | Y | [16] |
| DSCD | 238 kPa  <700% | - | 0.0359 | 0.52 | N | 9 h | N | Y | [17] |
| Hep-PDA-rGO | <300 kPa  <400% | <600 kPa  <60% | 3.63 | 0.24 (0-100%), 1.45 (100-350%) | N | - | Y | Y | [18] |
| PAM@CNC/TA-Ag NC | 110 kPa  2000% | - | 0.56 | 1.02 | 334.9 ms | - | Y | N | [19] |
| EGSD-Zn^2+^/PHA-I | 30 kPa  730% | 550 kPa  95% | <0.15 | - | - | 5 min | Y | Y | [20] |

**Table S2** Preparation for the PAM/BA-Ag@PDA hydrogel.

| **Samples** | **AM (mg mL^-1^)** | **BA (mg mL^-1^)** | **Ag@PDA (mg mL^-1^)** |
| --- | --- | --- | --- |
| 10AM/0.5BA-0.5Ag@PDA | 100 | 0.5 | 0.5 |
| 10AM/0.5BA-1.0Ag@PDA | 100 | 0.5 | 1.0 |
| 10AM/0.5BA-2.0Ag@PDA | 100 | 0.5 | 2.0 |
| 15AM/0.5BA-0.5Ag@PDA | 150 | 0.5 | 0.5 |
| 15AM/0.5BA-1.0Ag@PDA | 150 | 0.5 | 1.0 |
| 15AM/0.5BA-2.0Ag@PDA | 150 | 0.5 | 2.0 |
| 20AM/0.5BA-0.5Ag@PDA | 200 | 0.5 | 0.5 |
| 20AM/0.5BA-1.0Ag@PDA | 200 | 0.5 | 1.0 |
| 20AM/0.5BA-2.0Ag@PDA | 200 | 0.5 | 2.0 |
| 10AM/1.0BA-0.5Ag@PDA | 100 | 1.0 | 0.5 |
| 10AM/1.0BA-1.0Ag@PDA | 100 | 1.0 | 1.0 |
| 10AM/1.0BA-2.0Ag@PDA | 100 | 1.0 | 2.0 |
| 15AM/1.0BA-0.5Ag@PDA | 150 | 1.0 | 0.5 |
| 15AM/1.0BA-1.0Ag@PDA | 150 | 1.0 | 1.0 |
| 15AM/1.0BA-2.0Ag@PDA | 150 | 1.0 | 2.0 |
| 20AM/1.0BA-0.5Ag@PDA | 200 | 1.0 | 0.5 |
| 20AM/1.0BA-1.0Ag@PDA | 200 | 1.0 | 1.0 |
| 20AM/1.0BA-2.0Ag@PDA | 200 | 1.0 | 2.0 |
| 10AM/1.4BA-0.5Ag@PDA | 100 | 1.4 | 0.5 |
| 10AM/1.4BA-1.0Ag@PDA | 100 | 1.4 | 1.0 |
| 10AM/1.4BA-2.0Ag@PDA | 100 | 1.4 | 2.0 |
| 15AM/1.4BA-0.5Ag@PDA | 150 | 1.4 | 0.5 |
| 15AM/1.4BA-1.0Ag@PDA | 150 | 1.4 | 1.0 |
| 15AM/1.4BA-2.0Ag@PDA | 150 | 1.4 | 2.0 |
| 20AM/1.4BA-0.5Ag@PDA | 200 | 1.4 | 0.5 |
| 20AM/1.4BA-1.0Ag@PDA | 200 | 1.4 | 1.0 |
| 20AM/1.4BA-2.0Ag@PDA | 200 | 1.4 | 2.0 |
| 10AM/2.0BA-0.5Ag@PDA | 100 | 2.0 | 0.5 |
| 10AM/2.0BA-1.0Ag@PDA | 100 | 2.0 | 1.0 |
| 10AM/2.0BA-2.0Ag@PDA | 100 | 2.0 | 2.0 |
| 15AM/2.0BA-0.5Ag@PDA | 150 | 2.0 | 0.5 |
| 15AM/2.0BA-1.0Ag@PDA | 150 | 2.0 | 1.0 |
| 15AM/2.0BA-2.0Ag@PDA | 150 | 2.0 | 2.0 |
| 20AM/2.0BA-0.5Ag@PDA | 200 | 2.0 | 0.5 |
| 20AM/2.0BA-1.0Ag@PDA | 200 | 2.0 | 1.0 |
| 20AM/2.0BA-2.0Ag@PDA | 200 | 2.0 | 2.0 |

[1] Z. Tian, Z. Zhu, S. Yue, Y. Liu, Y. Li, Z. Yu, D. Yang, Nano Energy **2024**, 122, 109276.

[2] Y. Li, D. Yang, Z. Wu, F. L. Gao, X. Z. Gao, H. Y. Zhao, X. Li, Z. Z. Yu, Nano Energy **2023**, 109, 108324.

[3] R. Zhu, Y. Zheng, Q. Zhang, C. Yu, Z. Zhang, M. Huo, Adv. Funct. Mater. **2024**, 34, 2313155.

[4] J. Wang, Y. Bi, J. Liang, Z. Lu, K. Liu, Y. Liu, C. Jiang, Z. Yu, K. Zhang, X. Peng, K. Dong, Y. Xia, Nano Energy **2024**, 124, 109500.

[5] C. Liu, Y. Mao, L. Jiang, Q. Hu, Y. Zhang, F. Zhao, E. Zhang, X. Sun, Chem. Eng. J. **2024**, 482, 148863.

[6] H. Li, C. B. Chng, H. Zheng, M. S. Wu, P. J. Da Silva Bartolo, H. J. Qi, Y. J. Tan, K. Zhou, Adv. Sci. **2024**, 11, 2305702.

[7] A. Chae, G. Murali, S. Y. Lee, J. Gwak, S. J. Kim, Y. J. Jeong, H. Kang, S. Park, A. S. Lee, D. Y. Koh, I. In, S. J. Park, Adv. Funct. Mater. **2023**, 33, 2213382.

[8] G. Mogli, M. Reina, A. Chiappone, A. Lamberti, C. F. Pirri, I. Roppolo, S. Stassi, Adv. Funct. Mater. **2024**, 34, 2307133.

[9] N. Salimiyan, M. Gholami, R. Sedghi, Chem. Eng. J. **2023**, 471, 144648.

[10] X. Dai, Y. Wu, Q. Liang, J. Yang, L. B. Huang, J. Kong, J. Hao, Adv. Funct. Mater. **2023**, 33, 2304415.

[11] S. Yuan, J. Bai, S. Li, N. Ma, S. Deng, H. Zhu, T. Li, T. Zhang, Adv. Funct. Mater. **2024**, 34, 2309626.

[12] H. Zheng, M. Chen, Y. Sun, B. Zuo, Chem. Eng. J. **2022**, 446, 136931.

[13] Z. Nie, K. Peng, L. Lin, J. Yang, Z. Cheng, Q. Gan, Y. Chen, C. Feng, Chem. Eng. J. **2023**, 454, 139843.

[14] X. Yao, S. Zhang, L. Qian, N. Wei, V. Nica, S. Coseri, F. Han, Adv. Funct. Mater. **2022**, 32, 2204565.

[15] X. Yu, H. Zhang, Y. Wang, X. Fan, Z. Li, X. Zhang, T. Liu, Adv. Funct. Mater. **2022**, 32, 2204366.

[16] H. Zhang, H. Hu, Y. Dai, L. Xin, Q. Pang, S. Zhang, L. Ma, Acta Biomater. **2023**, 167, 348.

[17] A. Shit, S. Park, Y. Lee, B. Ryplida, N. Morgan, Y. C. Jang, E. J. Jin, S. Y. Park, Acta Biomater. **2023**, 171, 406.

[18] Y. Dou, Y. Zhang, S. Zhang, S. Ma, H. Zhang, J. Nanobiotechnol. **2023**, 21, 343.

[19] S. Hao, C. Shao, L. Meng, C. Cui, F. Xu, J. Yang, ACS Appl. Mater. Interfaces **2020**, 12, 56509.

[20] W. Wang, B. Jia, H. Xu, Z. Li, L. Qiao, Y. Zhao, H. Huang, X. Zhao, B. Guo, Chem. Eng. J. **2023**, 468, 143362.
